# Segregation within school classes: Detecting social clustering in choice data

**DOI:** 10.1371/journal.pone.0233677

**Published:** 2020-06-01

**Authors:** Fredrik Jansson, Gunn Elisabeth Birkelund, Mats Lillehagen

**Affiliations:** 1 Centre for Cultural Evolution, Stockholm University, Stockholm, Sweden; 2 Division of Applied Mathematics, Mälardalen University, Vasteras, Sweden; 3 Department of Sociology and Human Geography, University of Oslo, Oslo, Norway; 4 Institute for Analytical Sociology, Linköping University, Linkoping, Sweden; University of Arizona, UNITED STATES

## Abstract

We suggest a new method for detecting patterns of social clustering based on choice data. The method compares similar subjects within and between cohorts and thereby allows us to isolate the effect of peer influence from that of exogenous factors. Using this method on Norwegian register data, we address the question of whether students tend to cluster socially based on similar background. We find that common background correlates with making the same choices of curricular tracks, and that both exogenous preferences and peer influence matter. This applies to immigrant students from the same country, and, to some extent, to descendants of immigrants, but not to students from culturally similar countries. There are also small effects related to parents’ education and income.

## Introduction

With an increasing availability of large-scale data documenting people’s choices and behaviour, observations of people’s actual choices have become more accessible in a variety of situations. What these data typically do not measure directly, however, is information on the mechanisms behind these choices, such as the degree to which people interact and influence each other. We will here present a method for inferring propensities for peer influence between people based on similarity of choices, drawing inferences from administrative registry data from upper secondary school on students’ educational choices. We want to study so-called peer effects and school segregation at the micro-level; more specifically, we want to see if students of the same immigrant background within the same school and cohort influence each other’s choices of curricular tracks. The purpose is two-fold: to illustrate that it is indeed possible to trace out meaningful patterns of interaction in static data such as administrative registers, by suggesting a specific method, and to use that method to actually detect such patterns and address a substantial sociological issue. We will start by describing this issue and how it can be addressed using the presented method, before moving on to the details of the method.

### School segregation

Ethnic school composition is usually measured as the concentration of immigrant peers at school level [1, 2], or within educational cohorts within schools [3–8], or recently at several levels simultaneously, by including schools nested within school districts [9]. For immigrant students, ethnic school segregation means less exposure to the receiving society and fewer opportunities of learning the language [10]. These, and other, studies of segregation effects provide indirect support for the existence of peer influence, yet they lack data on social networks and what actually happens within the classrooms and in the school yard. Thus, the literature on school segregation has often *assumed* that social interaction takes place, yet, until recently, there were few quantitative studies on how students actually interact in school (see for instance [11]). Because direct information on social interactions will often not be available, and because registry and similar databases provide us with rich information contained within representative and reliable data on choices for a large number of cohorts and geographical areas, we aim at developing a method that allows us to infer clustering patterns at an aggregate level based on other variables, such as social background characteristics, using such data. Here, we use this method to assess the importance of (mainly) ethnic peer influence on the choice of curricular tracks in upper secondary schools.

The article employs Norwegian administrative register data with information on all upper secondary school students in five major cities over the years 2006–2011. Specifically, we will explore similarities in first year students’ choice of curricular tracks for their second and third year in upper secondary school. As we will explain, this is a choice that does not only have an educational impact, but also determines which students will remain classmates; it is thus also, to some degree, a choice of friends.

By studying students’ choices we aim at identifying peer effects between individuals who share the same socially significant attribute [12]. We focus mainly on ethnicity, but gender, age group and social background are other relevant examples. Most previous work has pointed to the fact that racial or ethnic homophily is one of the most important factors in friendship formation [12, 13], and one study suggests that segregation primarily takes place among students at the same grade level [14]. There are, however, other studies documenting that cross-ethnic friendships are also frequent [15].

Our main research question is whether we may infer peer influence on students’ choice of curriculum, based on the actual choices that we can observe. More specifically, we are interested in whether being and immigrant in general, immigrating from or having parents from a specific country, as well as originating in culturally similar countries is associated with peer influence on the choice of curricular specialisation in Norwegian upper secondary school. We suggest a novel method that can address this kind of questions and associated methodological challenges. We build our argument on the following logic:

1. Students have educational preferences before they start in upper secondary school. These preferences vary to some degree by students’ own characteristics, such as their immigrant status, gender and social background. Preferences are also likely to be affected by other factors, such as previous teachers, older students and other role models, and peer effects within lower secondary school and neighbourhoods. By studying curricular choices made by students in different educational cohorts at upper secondary school, we will document the strength of these exogenous preferences (as revealed in students’ educational choices).
2. Our main goal however, is to study choices induced by peer influence based on common social background characteristics, which can be considered a form of endogenous rather than exogenous preference formation. Students interact at school during their first year, and they may thereby influence each other’s educational choices. These social dynamics, revealed through patterns of social interactions, are likely related to students’ immigrant status, gender, social background, and other characteristics. Our main focus here is on immigrant status.
3. The challenge, then, is to differentiate between exogenous preferences and peer influence. We do so by comparing the two; that is, to assess the strength of peer influence during the first year at school we compare the educational choices within cohorts with the educational choices between cohorts. If the difference between the two are noticeable (as determined by a significance test), we interpret this difference as an outcome of endogenous preference formation due to social interaction at school.

Using this method, we will first explore potential peer influence effects related to immigrant background; second, we will see if these effects persist among descendants of immigrants, and, if so, if they apply to country of (parents) origin, or to some higher level of aggregation, such as cultural distance. Third, we additionally address other socio-economic factors, such as gender and parents’ education and income.

### Causes of segregation in school

There are mainly two reasons why we should expect to find evidence of social influence among students. First, individual opportunities and choices are often affected by social ties [16, 17]. In addition, individual choices affect others’ opportunities, as illustrated in Schelling’s [18] model on neighbourhood segregation; see also examples by [19–21]. Our study is limited to exploring the first part of these social dynamics. Given the importance of social ties, the vital question would then be with whom do we socialise?

Friendship formation is usually based on the well-documented preference for similarity in social relations [22–25]. At the group-level, it has been shown that status equality is essential for positive inter-group relations [26–28]. Associated with this argument is the notion of social distance, that is, the perceived affinity between people. When making choices, people prefer to conform to what other people with small social distance do [17]. In line with this, previous studies have shown that cross-ethnic ties are less stable [12, 29], and emotional support is more common in intra-ethnic ties [30]. We would thus expect homophilic dyadic interactions and friendship networks among students with similar characteristics, such as ethnic origin, and we would expect these interactions to bear some influence on students’ choices of educational specialisation [31] (see also [32]).

Second, homophily in friendship formation as well as perceived ingroup belonging may also be related to language. Language acquisition is closely related to social identification [33], and at school social ties are likely to be formed among immigrant students with similar language. Two German studies illustrate the crucial role of language [34, 35] in friendship formation, arguing that immigrants using the new language were more likely to befriend also native students (see also [36]). Thus, ethnic ingroup peer interaction as well as language proficiency are often strong predictors of ethnic identity [36], and immigrants of similar ethnic origin might prefer to stay together in class because they share a common ethnic identity. Theoretical modelling (e.g. [37]) has also supported the argument of ingroup preferences as a result of common references. Social interaction theory would thus lead us to expect social clustering of immigrant students from the same country of origin, and, from the same logic, we would expect less social interaction between descendants of immigrants, who, fluent in Norwegian, would be more likely to associate also with natives.

Third, immigrant students, including descendants of immigrants, may experience harassment, racism, and discrimination at school, which might contribute to ethnic friendship homophily. A number of studies in the US have addressed peer interactions among blacks and whites in school, showing that students from underprivileged backgrounds may feel less at a disadvantage when they are together with similar peers [38–48].

However, one study of ethnic stereotypes has shown a hierarchy, with “Norwegians and Swedes at the top, followed by Poles, and then Pakistani and Iraqi (Muslim) immigrants, with Somali immigrants and Roma people at the bottom” [49]. This ethnic hierarchy corresponds with cultural and geographical distance from Norway, and if students apply this ethnic ‘ranking’ on each other we might expect cultural distance to matter for their definitions of ingroups and outgroups.

### Social ties in school

During the last 15 years, and especially since the release of the CILS4EU dataset [50], a growing number of studies have addressed ethnically contingent social ties in European schools. When students are asked to list their friends, students of the same ethnicity are more likely to be on that list [30, 51, 52], and they also tend to be similar in cultural and socioeconomic characteristics [53]. Meanwhile, other data, collected in London, has suggested cross-ethnic friendship to be frequent and of high quality [15].

Here, we have a different approach, detecting consequences of intra-ethnic interaction and choice similarity that are revealed indirectly. We thereby utilise another type of measurement, complementing previous studies on self-declared ties. We thus aim to make both a substantial and methodological contribution to research on patterns of social exchange and their effects. Given that collecting data on stated preferences is costly, we often only have access to behavioural data, and the proposed method can make use of such already available data. Also, our results complement those derived from CILS4EU and other datasets, since we here detect preferences based on students’ actual behaviour, and thus avoid discussions about potential misreporting, boundary definitions and wishful thinking. Finally, when studying actual behaviour, the focus is on the consequences of social interaction—in this case educational choice—which is often what the investigator is mainly interested in.

### The present study

In the Norwegian school system, students in their last year of lower secondary school (at the age of 15) choose a study programme at upper secondary school for the next three years, either academic or vocational programmes. After the first year, they choose curricular specialisation tracks within their programme for years two and three. For example, a student who has chosen a general academic programme can specialise in natural sciences, social sciences or languages for years two and three. In general, the students on a specialisation track are a subset of the students on the whole programme. Typically, school classes, with a maximum of around 30 students, are formed within the specialisation tracks (with one or more school classes for each specialisation track dependent on the size of the student body). This means that students’ choice of specialisation track not only determines what subjects they will study, but also which of their classmates in the first year will most likely remain their classmates in the second or third year.

In the first year, students within the same class typically also engage in common activities such as eating, breaks, sports etc., which makes it very hard not to interact. Thus, students may coordinate their choices with their friends in order to remain classmates. Choices of specialisation tracks for the second and third year at school should therefore correlate with social ties within the class in the first year. We will distinguish between two mechanisms: individual-based choices related to common exogenous preferences on the one hand, and, on the other hand, choices related to peer influence (endogenous educational preferences and/or a preference for being together in class).

Using our data, we cannot differentiate between educational preference formation due to peer exposure in class and students making educational choices to maintain their social ties. There are recent methods to separate these mechanisms by means of a stochastic agent-based modelling approach [54, 55], but this approach requires rich and detailed longitudinal data on social ties. Whether choices result from maintaining social ties or from being influenced by the same peers, however, both are still effects from social clustering. Also, for our data, we do not have the problem of cause and effect: our observation is a choice that was made *after* a year of social exposure to peers.

In what follows, we will present the suggested method for inferring increased propensities for social clustering based on educational choices. We will then describe the data in further detail. The Results section first focuses on choices with respect to common country of origin (for both immigrants and descendants), robustness checks, and a discussion about how we should interpret effect sizes. We then proceed with broader measures than country of origin (e.g., through cultural distances), and also examine other demographic and socio-economic variables. Finally, we discuss the significance of the results and the potential use of the suggested method for further studies.

## Methods

We develop a three-step analysis to distinguish between the two mechanisms of exogenous educational preferences and choices related to endogenous (social and educational) preference formation that results from social interaction in class. We first explore similarity in first-year students’ choice of curricular tracks (within classes/educational cohorts). We expect these choices to be affected by their common exogenous preferences as well as endogenous effects. Second, we explore similarity in choices made by students that were not classmates (between classes/educational cohorts). This gives us a measure of common exogenous preferences between students making their choices in different years. Comparing choices within and between school classes, we aim at isolating choices related to within-class peer influence from choices related to exogenous educational preferences.

Our research question and data pose methodological challenges. For example, most linear and discrete choice models study choices in social isolation. There are relatively recent attempts at incorporating the impact of social networks and peer influence (e.g. [56]), but generally these assume a known network structure and include a measure of overall, instead of dyadic, peer effects (e.g., here it could be the classroom composition) on agents’ preferences or educational outcomes (for an overview, see [57]). Here, however, we are studying choices that are made simultaneously and the extent to which pairs of students make the same choices. Our unit of analysis is thus the dyad, which is, in turn, nested in a network structure.

We know of no existing alternative method that could directly address simultaneous dyadic choices, controlling for structural dependencies (and exogenous effects). For example, in a McFadden discrete choice model, the peer effects terms would end up being infinitely recursive (see also [58, 59]). Therefore, we suggest a combination of statistical methods, where, as will be described below, we study correlation coefficients between the existence of links (or ties/edges) in graphs constructed with respect to the input and output variable, respectively.

The method has been implemented in R, and the code is available on Github [60].

### Data requirements

We will illustrate the method using the specific case of choices of curricular tracks in school, but let us first, and more abstractly, provide the general assumptions for the kind of the data that can be used. The requirements increase the more that needs to be controlled for.

We are assuming data for a number of individuals where two variables are to be compared for whether similarity between two individuals in one of the variables is associated with similarity in the other, meaning that dyads are the units of analysis. More formally, the data could be construed as two two-mode matrices *A* and *B*, where the rows of the two matrices represent the same individuals and the columns the two variables, respectively. The aim is to compare the two one-mode matrices *AA*^*T*^ and *BB*^*T*^. We can control for structural dependencies using conventional methods (Quadratic Assignment Procedure, QAP, described below).

If data are grouped by a third variable, such that only individuals in the same group are to be compared with each other, then a summary measure can be computed using our combined meta-analytic QAP approach below.

If data are further grouped by a fourth variable, such that the groups of the third variable are subsets, then our method provides a way to measure the net effect of common membership (for pairs of individuals) in the subgroup defined by the third variable as compared to the group defined by the fourth. For a meaningful interpretation, all the subsets should be defined by common properties. The implications of demonstrating a significant such net effect is that properties that are exclusive to the subgroups as compared to the groups make individuals that are more similar along the first dimension more similar along the second.

### Measuring the effect size

The units of analysis are dyads, and each student in a class is paired once with each other student. This means that we have one matrix of the independent and one of the dependent variable, representing similarity between the students in each dyad. The matrices have the properties of adjacency matrices, and can each equivalently be represented by a graph: one graph where nodes represent students and links are drawn between students with the same characteristics, and another graph where the links represent similar educational choices. (Note that these are not social ties, but links indicating similarity between the nodes in a variable. Even though these are not necessarily social networks, they still have the mathematical properties of networks, and conventional methods apply.) Constructing these two graphs for all students in all classes, our research question is then whether the two types of graphs are correlated on the aggregate level. See Fig 3 for an example of what the two types of graphs can look like.

More formally, we have individuals *i* ∈ {1, 2, …, *n*}, each with an individual trait and a choice outcome. Let *v*_*i*_ be the individual trait, say, country of origin for individual *i*, and *V* = {*v*_1_, *v*_2_, …, *v*_*n*_} be the countries of origin for all *n* immigrant students in a school class. From this given data, we construct an adjacency matrix *A* such that *A*_*ij*_ = 1 if *v*_*i*_ = *v*_*j*_ and *A*_*ij*_ = 0 otherwise, that is, *A* indicates whether students *i* and *j* are from the same country. In this example, *A* is a matrix of binary variables, but it can also be generalised to a distance matrix, containing scale variables, where, for example, *A*_*ij*_ is a normed difference between *v*_*i*_ and *v*_*j*_. For the same individuals, we let *w*_*i*_ be the choice variable, say, choice of specialisation within a study programme, with *W* = {*w*_1_, *w*_2_, …, *w*_*n*_}. From this given data, we construct the corresponding adjacency matrix *B*, that is, *B*_*ij*_ = 1 if *w*_*i*_ = *w*_*j*_, students *i* and *j* made the same choice, and *B*_*ij*_ = 0 otherwise. The effect measure is the (element-wise) correlation *ρ*_*AB*_ between *A* and *B*.

Graph correlation coefficients can tell us whether having the same background, say, is associated with making the same choice. However, we are interested in whether such an outcome is caused by social exposure, or if it can be explained by higher propensities for students of given demographic or socio-economic characteristics to make certain choices. Another possible covariate is that going to certain schools may increase the probability of certain combinations, for example because a school may have profiled itself in a specific specialisation. In larger cities, schools are segregated by ethnicity and socio-economic status. These biases in school choice could affect the result. Thus, we need to control for both common exogenous educational preferences, related to demographic or socio-economic characteristics, and school-specific conditions.

The impact of both of these factors can be tested by comparing students not to their classmates, but to other students in the same programme and school, but who started in a different year. Instead of constructing one graph for each class (defined by school, programme and year), we have constructed a graph including all years (defined only by school and programme), with possible links between students only if they are not in the same class (so dyads of students from the same year are not included). This design allows for exogenous preferences and school-specific conditions to give correlations between the demographic or socio-economic variable and choice graph, while excluding within-class peer influence and choices based on retaining friends as classmates.

In more formal notation, we build adjacency matrices *A* and *B* over all cohorts {*C*_1_, …, *C*_*m*_} from the same school and programme, where all individuals belong to exactly one cohort. Values *A*_*ij*_ and *B*_*ij*_ are assigned as above, but for *i*, *j* ∈ *C*_*k*_, *k* ∈ {1, …, *m*}, *A*_*ij*_ = *B*_*ij*_ = *⌀*, that is, those values are excluded in the computation of *ρ*_*AB*_.

We thus perform a three-step analysis. First, we compute a *within-class* correlation coefficient, where students are compared pairwise to their classmates, with links between those in the same class with the same characteristics in the first graph, and those making the same choice in the second. This provides a measure of both endogenous and exogenous effects. Next, we compute a *between-class* correlation coefficient, where students are compared pairwise to everyone on the same programme in the same school, but in different cohorts (e.g., students starting in 2006 are compared to students starting in 2007–2011), thus measuring exogenous effects. Finally, we compare these two coefficients and measure the excess effect of demographic variables. To the extent that the exogenous effects are indeed of the same size in the first and second step, the excess effect isolates the endogenous effects, which we argue are effects of social exposure, which should be mainly driven by social ties (preferences for remaining together) and social influence from selected peers.

### Dealing with statistical dependencies

A complicating factor when studying choices in dyads is that we have strong dependencies, perhaps most evidently in what is known as *triad closure*. Given students A, B and C, if both B and C have made the same choice as A, then we know that B and C must also have made the same choice. This is the extreme, deterministic, case of a triad closure: it is not even possible to have exactly two links in a triad where the links signify having a trait in common. We need to control for the underlying graph structure. This can be done through the *Quadratic Assignment Procedure* (QAP), which is a strategy enabling statistical significance testing taking the graph structure into account [61].

In the QAP, the input graph is relabelled randomly, such that the characteristic under study retains its distribution, but is assigned randomly to all individuals. If, say, A and B have the same country of origin, then they will have a connection in the input graph. Depending on whether they made the same educational choice, they may have a connection also in the output graph. We retain the connections between nodes, but relabel them in the input graph, such that A and B may now be labelled C and D, say, and will be compared to these nodes in the output graph. Representing graphs as adjacency matrices, this amounts to randomly permuting the rows of the matrix and then applying the same permutation to the columns. The null hypothesis of the QAP test is that the observed correlation was drawn from the distribution of correlation coefficients on the set of all relabellings of the graphs. In practice, by repeating the relabelling procedure and computing simulated correlation coefficients, we can approximate this distribution and compare it to the observed correlation. The null hypothesis is rejected at significance level *α* if less than a fraction *α* of the simulated values are greater than the observed value.

Note that developing an alternative method based on an approach such as discrete choice modelling would also require similar simulations of different possible scenarios, with several design choices, in order to resolve the infinite recursion of the peer effects term.

### Summarising over classes

Conducting studies over several classes, it is not straightforward how to combine these into one single measure. Students over all schools are facing the same kind of choice, so both the independent and the dependent variables have the same meaning (e.g. a risk ratio of *r* means that students of the same origin are *r* times as likely as those from different origins to make the same choice). However, the effect sizes from each class are not directly comparable, as they depend on the graph structure and the variance. In order to estimate a summary effect, and to assess the consistency across classes, it is clear that the individual effect sizes need to be weighted. A conventional method is to weight them by their precision (measured by variance, e.g., small classes typically provide less confidence in the estimate of the actual effect than large classes). There is a method that also allows for the actual effects to vary between classes.

If we consider each class a separate experiment, then we can perform a *meta-analysis* over all experiments. In a *fixed-effect model*, this amounts to computing a weighted average of the effect sizes. The weights are commonly set to be the inverse variance of the effect in each study. Our studies fulfil the assumptions of samples being drawn from the same population, using the same variables etc.

There are, however, also sources of heterogeneity in that the graph structure and the distribution of educational choices vary over classes. Even if students from the same country would be equally more likely to choose similarly irrespective of school and class, the true effect size is still subject to structural limitations, varying between classes. In order to account for this, we mainly use a *random-effects model* instead, which reduces the differences in weights by adding to each within-study variance a random effects variance component measuring the variability between studies. For our main analysis, we also present the fixed effect sizes. In the present studies, the random effects variance is small, and there is thus little difference between the two models. For an overview of meta-analyses, we refer to [62].

The weighted mean effect *ρ* is computed as

$$\begin{array}{c} \rho =\frac{\sum_{i=1}^{k}w_{i}\rho_{i}}{\sum_{i=1}^{k}w_{i}}, \end{array}$$

where *k* is the total number of studies, *ρ*_*i*_ is the correlation coefficient for study *i*, and *w*_*i*_ the corresponding weight, computed as

$$\begin{array}{c} w_{i}=\frac{1}{\sigma_{i}^{2}+\tau^{2}}, \end{array}$$

where $\sigma_{i}^{2}$ is the within-study variance for study *i*, and *τ*^2^ is the between-studies variance, which is in turn estimated according to the [63] method, computed as

$$\begin{array}{c} \tau^{2}=\text{max}\{0,\frac{\sum_{i=1}^{k}v_{i}\rho_{i}^{2}-\frac{{\left(\sum_{i=1}^{k}v_{i}\rho_{i}\right)}^{2}}{\sum_{i=1}^{k}v_{i}}-\left(k-1\right)}{\sum_{i=1}^{k}v_{i}-\frac{\sum_{i=1}^{k}v_{i}^{2}}{\sum_{i=1}^{k}v_{i}}}\}, \end{array}$$

where $v_{i}=1/\sigma_{i}^{2}$ is the inverse within-study variance for study *i* (see also [62, p. 72–74], where *Y*_*i*_ is the effect size, *ρ*_*i*_). The computations are the same for a fixed-effect model, except for excluding the between-studies variance, which amounts to setting *τ*^2^ = 0.

Finally, we do not compute the variances and weighted mean directly over the correlation coefficients, but rather over their respective Fisher’s *z* transformed values [64, 65]. We then transform the weighted mean $\bar{z}$ back to the original scale. The *z* value of a correlation *ρ* is given by

$$\begin{array}{c} z=\frac{1}{2}\text{ln}\frac{1+\rho }{1-\rho }. \end{array}$$

In the extreme cases where *ρ* = 1 (which would happen mainly for simulated coefficients over small classes), we suggest to replace *ρ* by *ρ* − *ε*, where, for example, *ε* = 0.0001. This had no observable effect in our study.

### Combining approaches

We will combine the two approaches for dealing with statistical dependencies and summarising over classes into what we can label a meta-analytic QAP approach. Following the idea of QAP, for a class *i* ∈ {1, …, *n*}, we simulate 2*m* graphs with the same graph structure, producing *m* simulated correlation coefficients *r*_1,*i*_, …, *r*_*m*,*i*_, and thus a probability distribution of the correlation coefficient under the null hypothesis of no effect in the given graph structure. From this we can estimate the variances $\sigma_{i}^{2}$ and thus also the between-study variance *τ*^2^. The inverse of the sum of the within- and between-study variances (*w*_*i*_ from the previous section) of this distribution then gives us the weight applied to the actual correlation coefficient, *ρ*_*i*_, and the weighted sum *ρ*.

The aggregated correlation coefficient *ρ* needs to be compared to an aggregated distribution of simulated coefficients. Using the first simulated correlation coefficient *r*_1,*k*_ from each class *k* ∈ {1, …, *n*}, we can also use the derived variances to compute a weighted average simulated correlation ${\bar{r}}_{1}$. Reiterating this process for all of the simulated graphs for each class produces *m* weighted average correlations ${\bar{r}}_{1},\ldots ,{\bar{r}}_{m}$, and thus a probability distribution of possible meta-analytic correlations, given the graph structures of all the classes. Again, in line with the QAP approach, we use this distribution to test the weighted average of actual correlations against the null hypothesis that the correlation may have been caused by the graph structure only, with random allocation of choices preserving the distribution.

### Ethics statement

No new data has been collected. Access to the register data has been approved by The Norwegian National Committee for Research Ethics in the Social Sciences and the Humanities (NESH), the NSD Data Protection Services (Personvernombudet) and Statistics Norway.

## Data

We used Norwegian register data for students starting their upper secondary education in the years 2006–2010, and starting their curricular specialisation the following year, in five Norwegian cities and 120 schools. We limit the number of cohorts to only these five, to maintain similarity for the between-cohort comparisons. Henceforth we refer to the students in a programme at the same school within the same year as a class (even though, in reality, this may correspond to several school classes if there are many students on the same programme within the same school).

The total number of individuals in the data is 51,315. We used only students starting their curricular specialisation track one year after they started at the general programme, and at the same school, leaving 42,577 individuals in our data, out of which 2,940 (6.9%) are immigrants and 6,847 (16%) are native-born descendants of immigrants. Among the descendants, 4,675 (11%) have an immigrated mother. We included only students whose country of origin is known.

The number of individuals, *N*, included in the respective analyses varies, for several reasons. In general, *N* is larger in the between- than the within-class analyses of ethnicity. In order to compute a correlation coefficient for choices with respect to shared origin, there needs to be at least one pair of students from the same country (and one pair from different countries) in the same class. When comparing over several years, it is more likely that this condition will be fulfilled. Note also that, at the same time, the number of classes, *n*, is smaller when classes are defined as including several years. We performed a robustness check, described in the Appendix section Alternative designs, to investigate whether the slightly different subsetting of data affected the correlation coefficient.

The variable under study also impacts *N*. For our scale variables: cultural distances, parental education and parental income, there needs not be a pair of students of shared origin in order to compute a measure, which enables a larger *N*. At the other end, *N* is reduced by the fact that the educational level of parents of immigrants is not always known, and we can only compute cultural distances between pairs of students from countries included in the World Values Survey.

As described in the Introduction, students first choose a general programme for their first year at upper secondary school, and then a curricular specialisation track within the programme for their second and third year. The most popular choice is a general academic programme, followed by 23,636 (56%) students in our data. Among these students, almost everyone chose a specialisation in either natural sciences (10,816, or 46%) or language, social sciences and economy (12,264, or 52%). The remaining students not on a general study programme followed a variety of mainly vocational tracks. The number of study programmes in the data is 22, of which 14 had at least 100 students. The number of specialisations is 53, of which 32 had at least 100 students. The most common choices of specialisation tracks and origins of immigrants and descendants are presented in the Appendix section Origins and choices of the students.

## Results

Our main study investigates the extent to which pairs of immigrant students within the same class (students at the same school, programme and year) make the same choice of curricular track, dependent on whether they also share country of origin. We compare this result to the choices made by students on the same school and programme, but who were in other cohorts (i.e., between cohorts). We also compare within- and between-cohort choices among descendants of immigrants, and then go on to investigate different levels of origin. Is peer influence more prevalent between students from similar countries, and do we find this pattern also when considering immigrants as one group versus natives? Finally, we explore effects related to other demographic and socio-economic variables, such as gender, and parents’ education and income. We present robustness checks in conjunction with the results. To further calibrate the robustness of our method, we have also investigated alternative designs, which are presented in the Appendix.

All the weighted correlations and p-values from our studies are summarised in Table 1. In the following subsections, we describe the tests and their results in more detail.

**Table 1:** Summary of results. Correlation coefficients, significance levels and p-values (from the QAP, without adjustment for multiple testing in subgroups) for all 16 separate tests.

Test	Group	Subgroup	Within		p	Betw.		p	Diff.		p
***Ethnicity***	*Im*.	*All*	.057	***	.000	.019	**	.004	.038	**	.006
		*Men*	.079	*	.016	.038	*	.047	.040		.090
		*Women*	.082	***	.000	.036	*	.011	.045	*	.012
	*Desc*.	*All*	.031	***	.000	.015	**	.001	.016	*	.033
		*Men*	.024	*	.043	.016	*	.047	.008		.232
		*Women*	.049	***	.000	.031	***	.000	.018		.076
***Cult. dist***.	*Im*.	*All*	.012		.304						
		*Men*	.018		.423						
		*Women*	.028		.241						
***Im. status***	*All*		.014	***	.000	.011	***	.000	.003		.251
***Sex***	*All*		.029	***	.000	.024	***	.000	.004		.052
	*Im*.	*All*	.029	*	.025	.004		.144	.025		.081
***Education***	*All*		.018	***	.000	.008	***	.000	.010	***	.000
	*Im*.	*All*	.022		.223	-.015		.503	.037		.227
***Income***	*All*		.009	***	.000	.002	**	.004	.008	*	.015
	*Im*.	*All*	.024	*	.011	-.006		.083	.030		.172

### Same country versus different countries of origin

Our main hypothesis is that students with the same country of origin will influence each other’s choices, so that students with the same country of origin make more similar choices than students with different countries of origin. We start by looking only at immigrant students within the same class. For each cohort, we constructed 1,000 simulated graphs for significance testing, following the concept of QAP.

**Fig 1:**
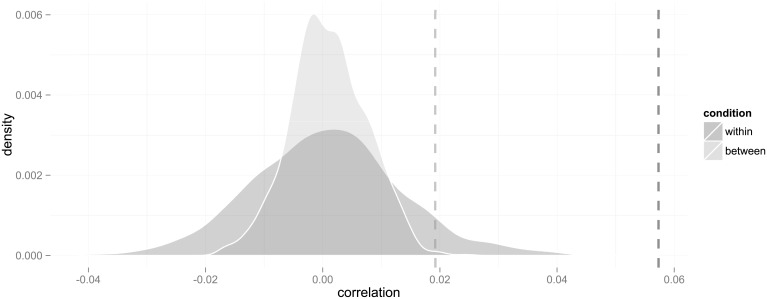
Probability distributions of correlations in the within- and between-group designs under the null hypothesis that choices are randomly allocated irrespective of country of origin. The dashed lines represent the observed correlations.

The weighted average correlation coefficient within classes is *ρ*_*w*_ ≈ 0.057 (or *ρ*_*w*_ ≈ 0.053 in the fixed-effect model). The probability under the null hypothesis to obtain this value or higher is *p* < 0.001. Thus, the null hypothesis can be rejected with high confidence (see the within condition in Fig 1), and we conclude that there is a significant correlation among immigrants between shared country of origin and making the same educational choice. These analyses are based on *n* = 125 classes, at 52 schools, with *N* = 1, 175 students. Thus, within each class, the average number of immigrants is 9.4, and each class includes, on average, immigrants coming from 7 (6.95) countries. In total, the numbers of shared origin dyads, triads etc. are 162, 32, 15, 3, 3, 0, 0, 1.

To what extent can this result be attributed to exogenous effects? We performed the same analysis, but between classes, that is, with graphs consisting of cohorts of all classes on the same programme in the same school over all years, removing links and non-links between students enrolled in the same year from the analysis.

The correlation in this analysis is *ρ*_*b*_ ≈ 0.019 (or *ρ*_*b*_ ≈ 0.023 in a fixed-effect model). This coefficient is smaller than within classes, but significantly different from random allocation of choices retaining the graph structure, with *p* < 0.01 (see the between condition in Fig 1). These analyses are based on *n* = 85 classes with *N* = 1, 964 students; the average number of immigrants is 23.1 from 13.8 countries. In total, the numbers of shared origin dyads, triads etc. are 242, 83, 33, 23, 6, 6, 6, and 7 groups are larger than 8.

How do these values compare, then, net of their respective graph structures? Pairing up the values from the simulated within- and between-class graphs randomly gives us a distribution of differences to which we can compare the actual difference. We find that, in more than 99% of the cases, the difference between the within- and between-class correlation coefficients are larger than the differences between the simulated values, which we accept as statistically significant (*ρ*_*w*_ − *ρ*_*b*_ ≈ 0.038, *p* < 0.01).

In this design, we do not control for other demographic variables. Particularly, girls and boys tend to segregate in classroom situations. Assuming there is no gender bias associated with certain ethnicities, there is no reason to expect gender to be a driving factor behind our results. However, we can test our hypothesis net of gender effects by performing the analyses on boys and girls separately. The effect is more significant for girls than for boys, and while there is a retained difference between the within- and between-class measures for girls, we could not safely conclude that there is a real difference for boys (see Table 1). These results show that the measures increase in absolute terms, while they also become less significant. However, samples are relatively small, which may account for higher p-values, but also, due to the structural dependencies in the data, the measures are not directly comparable.

As a robustness check, we also tested alternative designs, presented in the Appendix, where we make use of data from the natives in the class, or include only a subset of the students in the within-class condition for the between-class condition. These alternative designs and samples produced qualitatively similar results, and the remaining presented findings are based on our first meta-analytic QAP approach, which requires less computational power than the first alternative design, and includes more data than the second.

#### Descendants of immigrants

**Fig 2:**
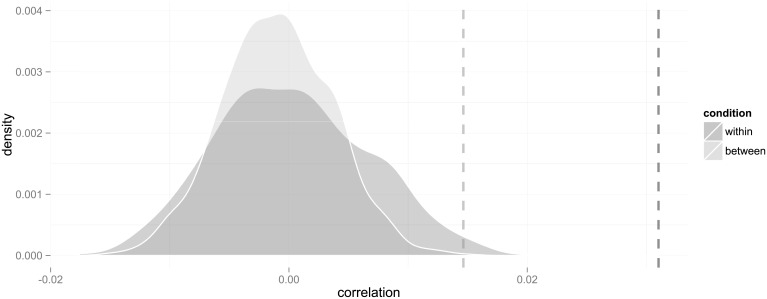
Probability distributions of correlations in the within- and between-group designs under the null hypothesis that choices are randomly allocated irrespective of mother’s country of origin. The dashed lines represent the observed correlations.

We performed the same analysis on native students with an immigrant mother and compared pairs of students whose mother came from the same country to pairs where their mothers came from different countries. The within-class measure is *ρ*_*w*_ ≈ 0.031 (*p* < 0.001, *N* = 2, 878, *n* = 205), while the between-class measure is *ρ*_*b*_ ≈ 0.015 (*p* ≈ 0.001, *N* = 3, 561, *n* = 89) (see Fig 2) and the difference *ρ*_*w*_ − *ρ*_*b*_ ≈ 0.016 (*p* ≈ 0.033). Similar to the immigrant analyses above, the effect seems to be mainly driven by women. Restricting the analysis to students where both parents come from the same country produces similar results.

Comparing the effect size for descendants to that of immigrants in the same way as computing the difference between the within- and between-class measures, gives a significant net measure of 0.026 (*p* ≈ 0.041) within classes (but not between classes). Thus, we found evidence that endogenous preference formation also remained in the second generation, though with a reduced effect.

#### Interpreting the effect size

How should we interpret the sizes of the correlation coefficients found here? First, it needs to be noted that the theoretical maximum is considerably below 1. We hypothesise that students of the same origin are more likely to choose similarly. However, a large correlation coefficient would require not only that all students from the same country make the same choice, but also that students from different countries always choose differently, which is not possible (and not predicted by the hypothesis) given the small number of available choices related to the number of students.

To get an estimate of a maximal effect size, we changed the specialisation choices of the students in such a way that all students from the same country always made the same choice. More specifically, within each class, everyone within a group was registered with the majority choice of their group. (In case of a tie, that choice was randomly selected among the most common ones.) This pattern provided a correlation within classes of *ρ*_*w*_ ≈ 0.29 and between classes of *ρ*_*b*_ ≈ 0.23. Thus, when everyone with the same background characteristics make the same choice, the maximum within-class effect size is 0.29.

**Fig 3:**
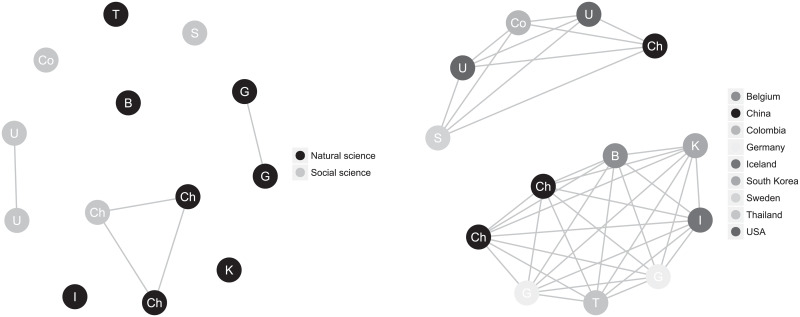
Example of a class from the data, with origin and choice graphs with correlation coefficient 0.059. The left panel has nodes coloured according to choice and connections between students of the same origin. The right panel has nodes coloured according to origin and connections between students making the same choice.

To build up an intuition for the magnitude of the effects found here, Fig 3 presents the origin and choice graphs for a typical class in the data. This class has a correlation (*ρ* ≈ 0.059) that is close to our weighted mean (*ρ* ≈ 0.057). This is a class on the academic programme, with natural science and social science as the two available choices of specialisation. By making only one change, so that all the Chinese students would choose natural science instead of one of them choosing social science, the choices would be completely in line with our hypothesis, and provide a theoretical maximum similar to the hypothetical discussion above. Such a change would produce a maximal effect size of *ρ*_max_ ≈ 0.24, which is also close to the maximum of the meta-analysis.

**Fig 4:**
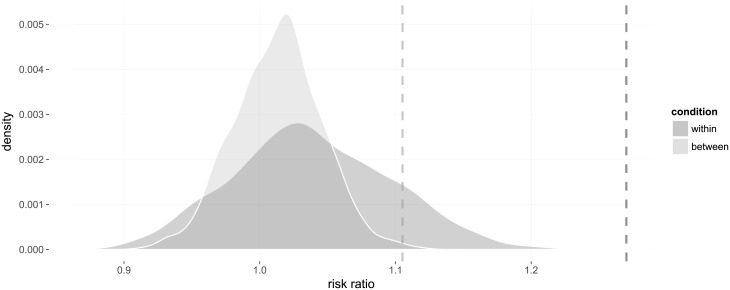
Probability distributions of relative risks in the within- and between-group designs under the null hypothesis that choices are randomly allocated irrespective of country of origin. The dashed lines represent the observed risk ratios.

The method is agnostic to choice of measure of effect size. Correlation coefficients have the benefit of being applicable also to continuous data, which is relevant for our analyses below. In the present case, however, the variables are dichotomous, and we could use other measures, such as ratios. Since several classes lack students from the same country making different choices, risk ratios are more viable (and easier to interpret) than odds ratios. The within-class ‘relative risk’ for immigrants from the same country to make the same choice compared to immigrants from different countries is RR_*w*_ ≈ 1.27 (*p* < 0.001, fixed effect RR_*w*_ ≈ 1.25, *p* < 0.001), that is, students sharing country of origin are 25% more likely to choose similarly (see Fig 4; cf. Fig 1). Between classes, the ratio is RR_*b*_ ≈ 1.11 (*p* ≈ 0.004, same fixed effect, *p* < 0.001). The additive difference between these risks is RR_*w*_ − RR_*b*_ ≈ 0.16 (*p* ≈ 0.016). However, it is easier to interpret the ratio of these risks, that is, the ratio of choosing more similarly among students of the same origin within versus between classes. Calculating this ratio, we get RR_*w*_/RR_*b*_ = log RR_*w*_ − log RR_*b*_ ≈ 1.15 (*p* ≈ 0.032, fixed effect RR_*w*_/RR_*b*_ ≈ 1.13, *p* ≈ 0.045). Similar to the correlation coefficients, all cases are significant, though at a lower level when comparing the within- to the between-classes measures.

### Correlations with cultural distances

In the previous analyses, students were binary categorised as belonging to the same or different groups. To further explore choice patterns related to immigrant students’ country of origin we have performed analyses where we allow for a continuous categorisation, using a “cultural distance” measure from the World Values Survey. Cultural distance, generated from the results of [66], is a measure of how far apart countries are, as documented by survey data on the populations’ attitudes to survival versus self-expression values and traditional versus secular–rational values. This measure allows us to explore, for example, if immigrant students from Sweden and Denmark are more similar in their educational choices than immigrant students from Sweden and Pakistan, and whether social choices are more likely between the first pair of students. We use the same methods as above, with the only difference being that the graph representing the independent variable is now weighted (i.e., the adjacency matrix has the values of the cultural distances instead of 0 and 1).

For each pair of students, we measured the cultural distance between them, based on their countries of origin, as the independent variable, and their educational choice, as the dependent variable. We excluded pairs of students with zero cultural distance, that is, those of common origin. The result was a correlation of *ρ*_*w*_ ≈ 0.012 (*N* = 1, 099, *n* = 175, *p* ≈ 0.30). Cultural distance thus does not seem to be a strong predictor of similarity in curricular choices. We also performed the analysis including pairs of students of the same origin. The result was a smaller effect than that of dichotomously defined within- and between-group pairs based on country of origin, suggesting that students do not on average choose more similar to students from countries that are culturally close than to other students, at least not by this measure of cultural distance.

### Immigrants versus natives

**Fig 5:**
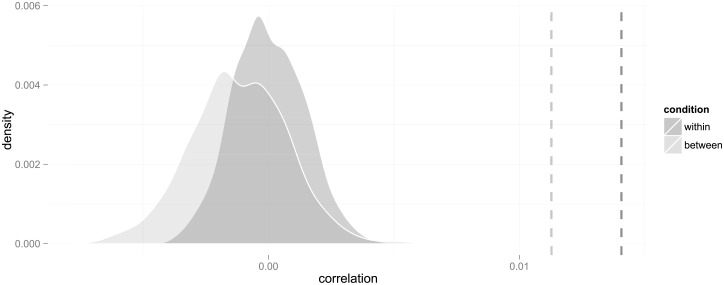
Probability distributions of correlations in the within- and between-group designs under the null hypothesis that choices are randomly allocated irrespective of whether the pairs of students are both immigrants or natives or one is an immigrant and the other a native. The dashed lines represent the observed correlations.

We performed an analysis where all immigrant students were grouped together and where we also included the natives. A pair of students are considered to belong to the same group if they are both natives, or both immigrants. The within-class correlation is then *ρ*_*w*_ ≈ 0.014 (*N* = 30, 300, *n* = 580, *p* < 0.001). The between-class correlation is *ρ*_*b*_ ≈ 0.011 (*N* = 33, 202, *n* = 174, *p* < 0.001) (see Fig 5). While the sample is large enough for these small effects to be statistically significant, the difference between them is not. This is consistent with our previous finding on cultural distance: similarity in curricular choices pertain to students from the same country of origin. We thus do no find any evidence of endogenous preference formation related to higher-order levels of shared origin, such as culturally similar countries, or being immigrants (regardless of country of origin), as compared to being natives.

### Other demographic and socio-economic variables

We also investigated the impact of gender, parental education and parental income. Restricting the analysis to immigrants gives us a good opportunity to check for potential confounding factors in our main results. After this, by including all students (i.e., also natives), we extended the analysis beyond ethnicity to see whether similar clustering takes place also for other individual characteristics.

We chose number of years of father’s education as the measure of parental education, and the average income of both parents as the income measure. If we lack information on one of the parents’ income, then the average is simply the other parent’s income.

While gender is a straightforward dichotomous variable, years of education and income are scale variables. In these latter cases, then, we calculated the distance between each pair of students by taking the absolute difference between the respective measures. The results are presented in Table 1.

Restricting the analysis first to the group of immigrants, the dataset is comparable to those in our previous studies. We find that there are no significant effects in the between-class analyses, nor in the differences between the within- and between-class correlations. Within classes, the effects are significant at the 0.05 level for gender and income, but not for education. Thus, our results are mainly inconclusive as to whether there are discernible effects among immigrants with respect to these demographic variables, which contrasts the demonstrated effects of country of origin. We conclude that the ethnicity effect cannot be explained by gender, parental education or parental income, and that the ethnicity effect appears to be more important.

Performing the same test, but now including all students (i.e., also natives), we find that all effects are significant. Again, the samples are obviously considerably larger (29,667–34,374 students, compared to 1,012–2,037 students with immigrant background). With such a large sample, the simulated probability distributions for the three different variables are highly similar, making the correlation coefficients roughly comparable. The largest coefficient is that for gender; we have two thirds of that effect for father’s education, and one third for parental income. At the same time, most of the effect for gender seems to be explained by common exogenous preferences, while there is some evidence of endogenous preference formation based on common parental education, and, possibly, also parental income.

For robustness, we also tested the influence of varying class sizes and number of available educational choices. We conclude from the results in the Appendix on robustness that the results are largely robust and that the major effects we have found are not dependent on the choice of including or excluding small classes with fewer choices.

## Conclusions

### Summary

In this paper, we suggest a new method for identifying endogenous preference formation based on specific characteristics. The method can be applied when we have large amounts of data that allow for a ‘control’ and ‘effect’ design. The studies are correlational, and we do not reconstruct actual social ties, but we identify added propensities for social clustering based on common characteristics in other variables.

We used this method to explore segregation at the micro-level, that is, we have analysed students within and between and school cohorts, to detect patterns of peer influence. In particular, we have explored if students make more similar choices of curricular specialisation to peers of the same country of origin as compared to other students, and whether this is a result of endogenous preference formation in classes. To do so, we differentiate between (a) exogenous preferences, that is, choices related to common inherent preferences (such as country-specific preferences, and going to the same school), and (b) peer influence, that is, endogenous preferences related to social processes within the class, as well as preferences for being together in class next year. We are, however, not able to differentiate between the two last types of endogenous mechanisms, which we refer to as peer influence. Table 1 summarises our main findings.

Comparing within and between educational cohorts, our results show that immigrant students’ educational choices correlate with common country of origin, and, to some extent, this is also the case for descendants of immigrants, categorised by their mother’s country of origin. The differences between these measures, within and between cohorts, are also significant, giving us a measure of endogenous preference formation, which is the net effect that measures peer influence in class. The effect is possibly larger among female students. Further, the effect is significantly larger within classes for immigrants than descendants, but not between classes, which suggests that there is stronger ethnic clustering in the choices made by immigrants than descendants, while preferences from home may be similar.

We also investigated whether the domain can be extended to include pairs of students from culturally similar countries, but categorising students by cultural distances was not predictive for educational choices. This result is largely consistent with what [67] found.

Finally, we compared all immigrants with all natives. While educational choices do correlate with being an immigrant, this correlation can be attributed to immigrants more often making similar choices in general rather than to peer influence. Again, social boundaries have often been found to be larger between some immigrant groups than between immigrant groups and natives [67].

From these observations, we draw the conclusion that there is clustering of peer influence among immigrant students, and to some extent descendants of immigrants, based on shared country of origin, and the results confirm that the level of analysis should be the country-level, as also suggested by [68].

While there is more evidence for these effects among female than male students, there appear to be no significant endogenous preference effects based on only gender, nor on having similar education or parents with similar income, in the group of immigrants. It should be noted that for example the group of girls in a class is large and that we are measuring average effects. It might be less likely that all girls influence each other than students of a more confined group, and the conclusion from this is not that gender is unimportant, but that it is not the right level of aggregation for peer influence.

While we found no significant effects on curriculum choice over and above exogenous effects, same gender has previously been shown to be important in social tie formation [52], and given the larger ethnic effect among girls, there may be an interaction effect. In addition, the observed patterns may potentially be confounded by other factors, such as school performance [55]. A previous study, however, using data on students in English, German, Dutch and Swedish schools, found that cultural and socioeconomic differences did not explain intra-ethnic homophily in friendship patterns [53]. As it currently stands, though, the presented method does not enable us to measure the effects from one variable directly controlled for another. For this purpose, we call for further methods development.

Looking at the whole population, including natives, vastly multiplies the number of observations. The results showed that there is a gender-based curricular choice similarity, but that these can be ascribed to common exogenous preferences. Looking at students’ social origin, however, we found some evidence of endogenous preferences based on similar background with respect to parents’ education and income.

In sum we interpret the differences between choice of curriculum within cohorts and choices between cohorts, as an indication of ethnic peer influence. More specifically, a graph over peer influence will more likely have cliques based on country of origin than the other categories we have investigated, such as gender. Comparing curricular choices within and between cohorts, we find on the aggregate scale that students behave more similarly based on their background. Our design shows that this is not likely to be exclusively an effect of having a common background as such. Rather, we would argue, students who are exposed to each other have the potential of affecting each other, and students with common social characteristics make similar choices to a greater extent than students of different backgrounds. We believe the simplest explanation to be an increased social exchange between the students.

**Fig 6:**
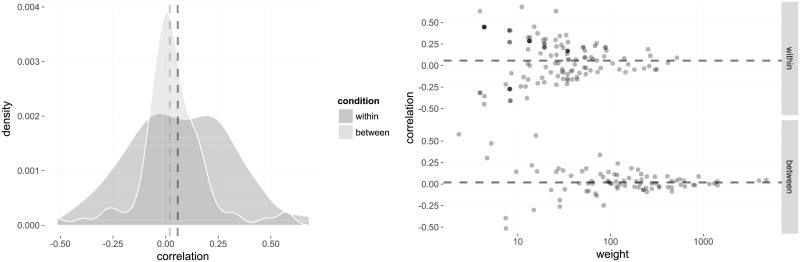
The left panel shows the distribution of correlation coefficients in each class (within) or programme/school combination (between). The right panel shows the coefficients plotted against their weights (on a log scale) in the meta-analysis. The dashed lines represent the weighted aggregated correlation coefficients from the meta-analysis.

Still, there are alternative explanations. One possibility is that other processes taking place in the classroom are driving the similarity in choices. One important example of this type of mechanisms, would be if individual teachers are having an effect on later choices within the class they are teaching. If this is the case, then the teacher effect would have to be minority-specific to explain our findings; that is, we would expect that teachers would influence students with the same ethnic background in ways that make their choices more similar. On the other hand, if teachers have a general effect on all their students, which we consider plausible, then the teacher effect would not increase the correlation coefficient or risk ratio for students of shared origin. Still, if teachers are in fact influencing their students in this way, then this will arguably lead to greater variation in effect sizes across different classes. We tested this, and Fig 6 in the appendix shows that while there is substantial variation, the effect sizes are less spread in classes with higher weight in the overall analysis. Another prediction from teacher effects, and other similar effects within cohorts, is that between-class correlations should be higher when comparing years close in time. (However, this could also be driven by substantial changes in school conditions over time, so it would not alone be evidence of teacher and similar effects.) We have measured the effects at a more disaggregated level in the Appendix section Disaggregated data, finding no clear pattern that between-class correlation coefficients drop off significantly when considering years further apart. This suggests that our results are not driven by a teacher effect, or other cohort-specific effects.

Another theoretical possibility is the existence of streaming, where students are administratively sorted into different classes based on their background characteristics. This is not likely to be important, as assigning students in secondary school based on ethnicity has been declared unlawful in the Norwegian school system, based on national and international discrimination laws (see e.g. an announcement by The equality and anti-discrimination ombudsman in Norway, ref. 12/186-10, www.ldo.no/globalassets/arkiv/uttalelser_pdf/2012/12_186.pdf).

Finally, these educational choices also determine who will be the students’ future classmates. Surveys and interview studies find friends to be an important [69] or even the most important factor [70, 71] in choosing upper secondary schools in Norway and Sweden. We would expect that choosing who will remain classmates in the second and third year at school should have an even stronger social component.

### Discussion

The mechanisms related to homophily and endogenous preference formation are important to document, yet often difficult to explore empirically. We have in this paper developed a method to identify such effects in individuals’ choices.

Our main results suggest some social clustering of immigrant students at school, based on country of origin (but not cultural similarity). This applies in particular to female immigrant students.

In the Introduction we suggested three relevant social mechanisms: homophily, language difficulties, and discrimination or harassment. Our findings are in line with the homophily mechanisms: small social distances and perceived affinity or nearness between people contribute to group-based similarities in educational choices. Here we found evidence of endogenous preference formation when groups were defined by country of (own or parents’) origin.

For immigrant students, language problems is also a likely explanation. Immigrant students who are weak in Norwegian but fluent in another common language may prefer to stay together in class. Descendants of immigrants born in Norway are likely to master both Norwegian and their mother’s language, and may therefore interact more equally with natives and each other, which is in line with a smaller effect for descendants of immigrants.

Finally, the premise of a resistance strategy is that majority students categorise minorities from particular countries of origin into outgroups based on stereotypes. If this was a dominant mechanism, however, then we would have expected to find more evidence of similar choices among immigrant students from countries of origin with small cultural distances (such as Muslims).

We would expect our approach to be relevant for other topics of investigation as well. One obvious example would be when students are choosing educational tracks later in their educational careers. Often students have to move geographically to attend higher educational institutions. If students want to stay together, then we would expect social clustering to be of relevance for their choice of institution, but not necessarily choice of study programme or discipline. If students have influenced each others’ preferences, then we would expect social ties to also influence their choice of study programme or discipline. In this case, it may thus be possible to also disentangle endogenous educational preference formation from choices based on friendship.

Generally, many decisions are affected by social clustering, also outside the educational domain (such as mobility within jobs or between firms, marriage decisions and families’ decisions on where to move). What is necessary for the proposed method to work is that the data allows for comparisons within and between cohorts, and where individuals within a certain setting or institution face an overlapping set of choices. One example could be co-workers who started in a firm at the same time and who face decisions of whether to stay or leave, as well as what type of firm to leave for, if applicable. Such mobility decisions have been found to be affected by peer influence based on shared characteristics in a similar way to what we find in the current study [72, 73]. The theoretically challenging task would be to carefully delineate under what social conditions we might expect social influence to be of direct relevance for an individual’s decision-making.

In this study, we have conducted separate analyses where choice is potentially dependent on a number of different independent variables. Further development of the method includes finding reliable measures for comparing effect sizes between different aggregated analyses and generalising it to allow for multivariate and multivariable models, for example to study gender as contrasts or isolating effects from socioeconomic variables.

## Appendix

### Origins and choices of the students

The twenty most common choices of specialisation tracks (including tracks on both academic and vocational programmes) over the five years in our study are given in Table 2. The included programmes are (with abbreviations): General programme (G), Mass media and communication (MMC), Health, social subjects and sports (HSS), Building and construction (BC), Electrical, mechanical and machines (EMM), Music, dance and drama (MDD), Trades and services (TS), Food production (FP), Arts and crafts (AC) and Service subjects (SS).

**Table 2:** Distribution of students over specialisation tracks. The twenty most popular specialisation tracks and their programme (abbreviated), with proportional share of students registered on each for each of the five years in our study. All figures are in percent, and include both natives and immigrants.

Specialisation	Programme	2007	2008	2009	2010	2011
*Language, Social Science and Business Administration*	*G*	28	29	30	30	28
*Science*	*G*	26	26	25	24	27
*Mass Media and Communication*	*MMC*	7	7	8	8	9
*Sports and Athletics*	*HSS*	5	5	5	5	4
*Construction*	*BC*	4	4	3	3	3
*Electrical Energy*	*EMM*	2	2	3	3	3
*Para-Medical and Care*	*HSS*	2	2	2	3	2
*Music and Entertainment*	*MDD*	2	2	2	3	2
*Social Work—Children and Adolescents*	*HSS*	2	2	2	2	2
*Retail Sales and Commercial Services*	*TS*	2	2	2	2	2
*Catering*	*FP*	2	2	2	2	1
*Arts and Crafts*	*AC*	2	2	1	2	2
*Theatre and Performance*	*MDD*	2	1	1	1	1
*Vehicle Repairs and Maintenance*	*EMM*	1	1	1	1	1
*Industrial Technology*	*EMM*	1	1	1	1	1
*Computer Science and Electronics*	*EMM*	1	1	1	1	1
*Hairdressing*	*SS*	1	1	1	1	1
*Climatic and Environmental Technology*	*BC*	1	1	1	1	1
*Household Economics*	*FP*	1	1	1	1	1
*Dance and Performing Arts*	*MDD*	1	1	1	1	1

It should be noted that 2007 is the first year after a school reform, and that for this year, language was a separate specialisation from social science and economy. We grouped these together in order to facilitate comparisons between years (this affected 209 students, of which 14 immigrants). Two specialisations, with education codes 301102 and 361203 (Norwegian Standard Classification of Education, 2000) had students almost exclusively in 2007, so we removed the 204 students (28 immigrants) registered on these from the sample. Finally, one code, 301116, is referred to as erroneous in the standard, so we removed also the associated 91 students (17 immigrants).

The origins of the students in our main study and their educational choices of specialisations, among the two most common ones, natural and social science, and other (there are too few students on each vocational track to report these separately) are presented in Table 3. The table also lists mother’s origin for descendants of immigrants.

**Table 3:** Origins of immigrants and descendants. The twenty most common countries of origin for immigrants and mother’s origin for native descendants, with proportional share in the population from the country, and share of students on the specialisation tracks natural science (N); language, social science and economy (S); and other (O), respectively. The total is the summed share from the twenty most common countries, and shares in the whole set of immigrants and descendants, respectively, on the respective specialisation tracks. Note that school classes with too few immigrants to perform our analyses have been excluded. Including the whole population, not only those in our analysis, would increase the figures for “other” specialisations. All figures are in percent.

#	Immigrants	%	N	S	O	Descendants	%	N	S	O
*1*	Iraq	7	35	34	30	Pakistan	15	42	41	17
*2*	Sweden	7	43	46	11	Vietnam	8	61	26	13
*3*	Pakistan	6	39	38	22	Sri Lanka	8	70	24	6
*4*	Afghanistan	6	32	19	49	Sweden	7	38	56	6
*5*	Somalia	5	28	22	50	Turkey	5	29	43	28
*6*	Bosn.-Herz.	4	28	60	11	Denmark	5	37	55	8
*7*	Russia	4	38	38	25	India	4	65	27	8
*8*	USA	4	31	64	5	USA	4	48	48	4
*9*	Poland	4	33	26	42	Morocco	4	23	49	28
*10*	China	4	48	14	38	Philippines	3	40	49	10
*11*	Iran	3	30	43	27	UK	3	47	46	8
*12*	Germany	2	38	55	7	Germany	3	41	53	7
*13*	Sri Lanka	2	62	14	24	Poland	2	44	48	8
*14*	Philippines	2	29	29	43	Iran	2	51	41	8
*15*	Kosovo	2	22	44	33	Somalia	2	38	47	16
*16*	South Korea	2	52	41	7	China	2	58	34	8
*17*	Denmark	2	50	46	4	Eritrea	1	20	78	3
*18*	UK	2	35	54	12	Macedonia	1	48	39	12
*19*	Vietnam	2	42	38	19	Chile	1	36	55	9
*20*	Ethiopia	2	32	32	37	Kosovo	1	34	47	19
	***Total***	***73***	***38***	***35***	***27***	***Total***	***81***	***45***	***45***	***10***

### Disaggregated data

The effect sizes should be compared to the simulated distributions for drawing conclusions. To give an overview, however, of what the disaggregated data looks like, the distribution of correlation coefficients in each class (i.e., not controlled for structural dependencies) is given in Fig 6. The within-class distribution has (nonweighted) mean *μ*_*w*_ ≈ 0.072 and variance $\sigma_{w}^{2}\approx 0.056$, and the between-class distribution has mean *μ*_*b*_ ≈ 0.033 and variance $\sigma_{b}^{2}\approx 0.027$. The figure also shows the effect sizes plotted against their weights in the meta-analysis. Even if the variability is high over all classes, the effect sizes are less spread for classes of high weight.

We have included five years of data to get a sizable dataset that can provide a stable measure not too dependent on a particular year, while at the same time excluding comparisons so many years apart that school conditions may have changed considerably (see e.g. [74]). Table 4 presents within- and between-class correlations for analyses of choices based on country of origin, and based on individual years and pairs of years, respectively. Note that the samples are fairly small (approximately 250 immigrant students per year).

**Table 4:** Correlation coefficients year by year between educational choices and country of origin. Effect sizes (and *p*-values are given year by year within classes and in pairwise comparisons between years between classes.

	witdin	2007	2008	2009	2010
2006	.055 (.021)	.010 (.081)	.006 (.047)	.004 (.278)	.003 (.221)
2007	.072 (.013)		.022 (.019)	.031 (.098)	.030 (.043)
2008	.053 (.071)			.022 (.122)	.020 (.068)
2009	.004 (.484)				.089 (.083)
2010	.078 (.003)				

If school conditions influencing the between-class effect change rapidly, then we would expect a clear pattern where the between-class coefficient drops off quickly, the further apart the years are in the between-class comparison. Such effects could include a restructuring of specialisations in large schools or special targeting towards students of a certain background, for example through teachers involved during a short time period. The year 2006 potentially hints at a decreasing effect (though only the between-class comparison to 2008 is significant, while that to 2007 is not). For the other years there is no clear such pattern, especially when taking level of significance into account.

There is no clear trend for the within-class correlation coefficients. We do not know of any explanations beyond random variation for the drop in 2009.

### Alternative designs

In the present analyses, the graphs studied include only immigrant students, and thus, the graph structures we control for do account for statistical dependencies, but since these are only subsets of the entire classes, we do not use all the data available. The reason for this is that natives constitute a large majority of the data, so including them in the effect measure would lead to an estimate mainly measuring links of natives versus links of a native and an immigrant. It is possible, however, to include natives when computing the variances used for weighting classes, but at the same time including only immigrants when computing the correlation coefficient. In practice, this means that we first include the entire class. Then we randomly permute nodes (as in QAP), and remove the natives from the graphs only before computing the correlation coefficient.

Using this design for our main study on country of origin produced similar results. The within-class measure is *ρ*_*w*_ ≈ 0.063 (*p* < 0.001), the between-class measure is *ρ*_*b*_ ≈ 0.024 (*p* < 0.001), and the difference *ρ*_*w*_ − *ρ*_*b*_ ≈ 0.039 (*p* < 0.01).

Making within-class comparisons of immigrants puts higher restrictions on the ethnic compositions in classes than making between-class comparisons, since, in order to compute correlations, there needs to be at least one pair of students from the same country. As a result, there are more students included in the between-class analyses. In case there would be reason to suspect data selection to influence the measures, we performed an analysis on the subset of students included in the within-class design. This produced almost exactly the same between-class measure as before, *ρ*_*b*_ ≈ 0.019 (*p* ≈ 0.019, *N* = 916). Note that the smaller *N* gives a higher p-value with the same correlation coefficient. Also, not every student is included in the analysis, since the restricted dataset does not always make it possible to do cross-year comparisons.

In conclusion, the alternative designs produce qualitatively similar results.

### Robustness to diversity between classes

**Fig 7:**
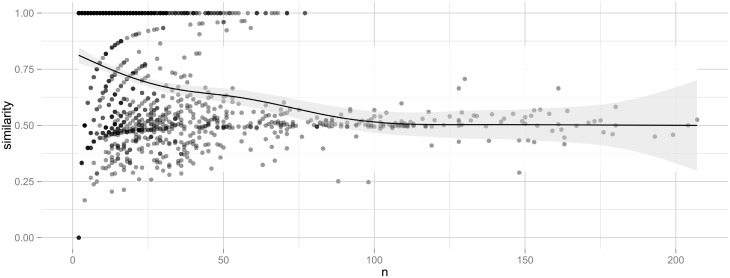
Proportion of pairs of students making the same educational choice with respect to class size.

Including all school classes in the analysis without restrictions, we have large diversity between classes with respect to available educational choices and overall popularity of each choice. Also, small classes are more homogeneous, in that a vast majority of the students make the same choice, probably due to a limited availability of different specialisation tracks. Our analytical design, utilising QAP and random-effects meta-analysis does allow for diversity between classes and weights are adjusted accordingly. Nevertheless, combining these approaches is a novel design not well documented in the literature, and as a robustness check, we repeated the analyses restricted to large schools, specifically to classes/educational cohorts within the same schools that had at least 50 students (75% of all classes in our analysis), including only students going to specialisation tracks with at least 10 students enrolled. The rationale for this threshold is that smaller classes are often more homogeneous within the group, with a large majority of students making the same choice, and vary more in the diversity in choices between the classes, thus making the classes less comparable. As can be seen in Fig 7, showing similarity in choices by class size, in smaller classes students often only have one choice (classes with a 100% similarity).

The results are similar to the cases where we included all classes. The major difference is that a few small effects from the previous studies are no longer significant. Such a change needs not be ascribed to structural differences in classes when including or excluding small classes, but is also consistent with the fact that the sample is smaller in the latter case.

Overall, the results change only little. The changes are that the ethnicity effects are no longer significant for men (*ρ*_*w*_ ≈ 0.054, *p* ≈ 0.091 and *ρ*_*b*_ ≈ 0.026, *p* ≈ 0.12 for immigrants, and *ρ*_*w*_ ≈ 0.015, *p* ≈ 0.12 and *ρ*_*b*_ ≈ 0.0098, *p* ≈ 0.10 for descendants), while the difference becomes significant for female descendants (*ρ*_*w*_ − *ρ*_*b*_ ≈ 0.025, *p* ≈ 0.035). The between-class coefficient with respect to income is no longer significant (*ρ*_*b*_ ≈ 0.00098, *p* ≈ 0.13), nor within classes for immigrants (*ρ*_*w*_ ≈ 0.016, *p* ≈ 0.064). Finally, the small differences with respect to sex, education and income for all students have changed their respective significance levels (with coefficients 0.0058, 0,0034 and 0.0063, and p-values 0, 0.041 and 0.0070, respectively).

It can be noted that most classes in our analysis are fairly large, since those are the ones with sufficient variation in the data. For 125 classes, 25% have fewer than 52 students, 50% have fewer than 98 and 75% fewer than 138. The mean is 97 students. It is possible in the largest cohorts that not all students have had opportunities to interact with everyone, which in those cases should make the test more conservative and bring the effect closer to the between-class coefficient.

## References

[1] Crosnoe R. Low-Income Students and the Socioeconomic Composition of Public High Schools. American Sociological Review. 2009;74:709–730. 10.1177/000312240907400502 21546987 PMC3086272

[2] Fekjær SN, Birkelund GE. Does the Ethnic Composition of Upper Secondary Schools Influence Educational Achievement and Attainment? A Multilevel Analysis of the Norwegian Case. European Sociological Review. 2007;23(3):309–323.

[3] Hanushek EA, Kain JF, Rivkin SG. New Evidence about Brown v. Board of Education: The Complex Effects of School Racial Composition on Achievement. Journal of Labor Economics. 2009;27:349–383.

[4] Hermansen AS, Birkelund GE. The Impact of Immigrant Classmates on Later Educational Outcomes. Social Forces. 2015;94:615–646.

[5] Szulkin R, Jonsson JO. Ethnic Segregation and Educational Outcomes in Swedish Comprehensive Schools In: SULCIS Working Paper Series. Stockholm: Stockholm University; 2007.

[6] Gould ED, Lavy V, Paserman DM. Does Immigration Affect the Long-Term Educational Outcomes of Natives? Quasi-Experimental Evidence. Economic Journal. 2009;119:1243–1269.

[7] Hardoy I, Schøne P. Does the Clustering of Immigrant Peers Affect the School Performance of Natives? Journal of Human Capital. 2013;7:1–25.

[8] Schwartz AE, Stiefel L. Immigrants and Inequality in Public Schools In: Duncan GJ, Murnane RJ, editors. Whither Opportunity? Rising Inequality, Schools, and Children’s Life Chances. New York: Russell Sage; 2011 p. 419–442.

[9] Leckie G, Goldstein H. A multilevel modelling approach to measuring changing patterns of ethnic composition and segregation among London secondary schools, 2001-2010. Journal of the Royal Statistical Society: Series A (Statistics in Society). 2015;178(2):405–424.

[10] Espinosa KE, Massey DS. Determinants of English Proficiency among Mexican Migrants to the United States. International Migration Review. 2014;31(1):28–50. 12320905

[11] Entorf H, Lauk M. Peer Effects, Social multipliers and Migrants at School: An International Comparison. Journal of Ethnic and Migration Studies. 2008;34(4):633–654.

[12] McPherson M, Smith-Lovin L, Cook JM. Birds of a Feather: Homophily in Social Networks. Annual Review of Sociology. 2001;27:415–444.

[13] Kandel DB. Homophily, Selection, and Socialization in Adolescent Friendships. American Journal of Sociology. 1978;84(2):427–436.

[14] Leszczensky L, Pink S. Ethnic segregation of friendship networks in school: Testing a rational-choice argument of differences in ethnic homophily between classroom- and grade-level networks. Social Networks. 2015;42:18–26.

[15] Bagci SC, Kumashiro M, Smith PK, Blumberg H, Rutland A. Cross-ethnic friendships: Are they really rare? Evidence from secondary schools around London. International Journal of Intercultural Relations. 2014;41:125–137.

[16] Granovetter M. The Strength of Weak Ties. American Journal of Sociology. 1973;78(6):1360–1380.

[17] Akerlof GA. Social Distance and Social Decisions. Econometrica. 1997;65(5):1005–1027.

[18] Schelling TC. Micromotives and Macrobehavior. New York, New York: W. W. Norton & Company; 1978.

[19] Manski CF. Identification of Endogenous Social Effects: The Reflection Problem. The Review of Economic Studies. 1993;60(3):531–542.

[20] Brock WA, Durlauf SN. Discrete Choice with Social Interactions. The Review of Economic Studies. 2001;68(2):235–260.

[21] Hedström P, Bearman P. The Oxford Handbook of Analytical Sociology. Oxford, United Kingdom: Oxford University Press; 2009.

[22] Moody J. Race, School Integration, and Friendship Segregation in America. American Journal of Sociology. 2001;107:679–716.

[23] Mouw T, Entwisle B. Residential Segregation and Interracial Friendship in Schools. American Journal of Sociology. 2006;112(2):394–441.

[24] Goodreau SM, Kitts JA, Morris M. Birds of a feather, or friend of a friend? using exponential random graph models to investigate adolescent social networks. Demography. 2009;46(1):103–125. 10.1353/dem.0.0045 19348111 PMC2831261

[25] Munniksma A, Scheepers P, Stark TH, Tolsma J. The Impact of Adolescents’ Classroom and Neighborhood Ethnic Diversity on Same- and Cross-Ethnic Friendships Within Classrooms. Journal of Research on Adolescence. 2017;27(1):20–33. 28498532 10.1111/jora.12248

[26] Allport GW. The Nature of Prejudice. Cambridge, Massachusetts: Perseus Books; 1954.

[27] Ahmed A. Group Identity, social distance and intergroup bias. Journal of Economic Psychology. 2007;28:324–337.

[28] Skvoretz J. Diversity, Integration, and Social Ties: Attraction versus Repulsion as Drivers of Intra- and Intergroup Relations. American Journal of Sociology. 2013;119(2):486–517.

[29] Aboud F, Mendelson M, Purdy K. Cross-race peer relations and friendship quality. International Journal of Behavioral Development. 2003;27(2):165–173.

[30] Baerveldt C, Van Duijn MAJ, Vermeij L, Van Hemert DA. Ethnic boundaries and personal choice. Assessing the influence of individual inclinations to choose intra-ethnic relationships on pupils’ networks. Social Networks. 2004;26(1):55–74.

[31] Manzo G. Educational Choices and Social Interactions: A Formal Model and a Computational Test. Comparative Social Research. 2013;30:47–100.

[32] Jæger MM. Economic and social returns to educational choices. Extending the utility function. Rationality and Society. 2007;19(4):451–483.

[33] Ochs E. Constructing Social Identity: A Language Socialization Perspective. Research on Language and Social Interaction. 1993;26(3):287–306.

[34] Titzmann PT, Silbereisen RK. Friendship Homophily Among Ethnic German Immigrants: A Longitudinal Comparison Between Recent and More Experienced Immigrant Adolescents. Journal of Family Psychology. 2009;23(3):301–310. 19586193 10.1037/a0015493

[35] Titzmann PT, Silbereisen RK, Mesch GS, Schmitt-Rodermund E. Migration-Specific Hassles Among Adolescent Immigrants From the Former Soviet Union in Germany and Israel. Journal of Cross-Cultural Psychology. 2011;42(5):777–794.

[36] Phinney JS, Romero I, Nava M, Huang D. The Role of Language, Parents, and Peers in Ethnic Identity Among Adolescents in Immigrant Families. Journal of Youth and Adolescence. 2001;30(2):135–153.

[37] Jansson F. What Games Support the Evolution of an Ingroup Bias? Journal of Theoretical Biology. 2015;373:100–110. 25794651 10.1016/j.jtbi.2015.03.008

[38] Coleman JS, Campbell E, Hobson C, McPartland J, Mood A, Weinfeld F, et al Equality of Educational Opportunity. Washington, D. C: U.S. Government Printing Office; 1966.

[39] Finkelstein NW, Haskins R. Kindergarten Children Prefer Same-Color Peers. Child Development. 1983;54(2):502–508.

[40] Sagar HA, Schofield JW, Snyder HN. Race and Gender Barriers: Preadolescent Behavior in Academic Classrooms. Child Development. 1983;54:1032–1040.

[41] Schofield JW, Sagar HA. Peer Interaction in an Integrated Middle School. Sociometry. 1989;40:130–138.

[42] Hallian MT, Williams RA. Interracial Friendship Choices in Secondary Schools. American Sociological Review. 1989;54:67–78.

[43] McClelland KE, Auster CJ. Public Platitudes and Hidden Tensions: Racial Climates at Predominantly White Liberal Arts Colleges. Journal of Higher Education. 1990;61(6):607–642.

[44] Feagin JR, Sikes MP. Living with Racism The Black Middle-Class Experience. Boston, Massachusetts: Beacon Press; 1994.

[45] Tatum BD. Why are all the black kids sitting together in the caferteria? And other conversations about race. New York: Basic Books; 1997. 10.1126/science.abn0865 34822268

[46] Clotfelter CT. Interracial Contact in High School Extracurricular Activities. The Urban Review. 2002;34(1):25–46.

[47] Conger D. Within School Segregation in an Urban School District. Educational Evaluation and Policy Analysis. 2005;27:225–244.

[48] Carter DJ. Why the Black Kids Sit Together at the Stairs: The Role of Identity-Affirming Counter-Spaces in a Predominantly White High School. Journal for Negro Education. 2007;76:542–554.

[49] Bye HH, Herrebrøden H, Hjetland GJ, Røyset GØ, Westny LL. Stereotypes of Norwegian social groups. Personality and Social Psychology. 2014;55:469–476. 10.1111/sjop.12141 PMC4282792 24975918

[50] Kalter F, Heath AF, Hewstone M, Jonsson JO, Kalmijn M, Kogan I, et al. Children of Immigrants Longitudinal Survey in Four European Countries (CILS4EU). GESIS Data Archive, Cologne, Germany; 2016. Available from: cils4.eu.

[51] Baerveldt C, Zijlstra B, de Wolf M, Van Rossem R, Van Duijn MAJ. Ethnic Boundaries in High School Students’ Networks in Flanders and the Netherlands. International Sociology. 2007;22(6):701–720.

[52] Kalter F, Kruse H. Ethnic diversity, homophily, and network cohesion in European classrooms In: Koopman R, Lancee B, Schaeffer M, editors. Social Cohesion and Immigration in Europe and North America: Mechanisms, Conditions, and Causality. London, United Kingdom: Routledge; 2015 p. 187–207.

[53] Smith S, Maas I, van Tubergen F. Ethnic ingroup friendships in schools: Testing the by-product hypothesis in England, Germany, the Netherlands and Sweden. Social Networks. 2014;39(1):33–45.

[54] Steglich C, Snijders TAB, Pearson M. Dynamic Networks And Behavior: Seperating Selection From Influence. Sociological Methodology. 2010;8:329–393.

[55] Lomi A, Snijders TAB, Steglich CEG, Torló VJ. Why Are Some More Peer Than Others? Evidence from a Longitudinal Study of Social Networks and Individual Academic Performance. Social Science Research. 2011;40(6):1506–1520. 10.1016/j.ssresearch.2011.06.010 25641999 PMC4309286

[56] Wichmann B, Chen M, Adamowicz W. Social Networks and Choice Set Formation in Discrete Choice Models. Econometrics. 2016;4(4):42.

[57] Benhabib J, Bisin A, Jackson MO, editors. Handbook of Social Economics. San Diego, California: Elsevier; 2011.

[58] Manski C, Wise D. College Choice in America. Cambridge, Massachusetts: Harvard University Press; 1983.

[59] Sacerdote B. Peer Effects in Education: How Might They Work, How Big Are They and How Much Do We Know Thus Far? In: Hanushek EA, Machin S, Woessmann L, editors. Handbook of the Economics of Education. vol. 3 Elsevier; 2011 p. 249–277.

[60] Jansson F. Metaanalytic QAP;. github.com/fredrik-jansson/metaqap.

[61] Krackhardt D. Predicting with Networks: Nonparametric Multiple Regression Analyses of Dyadic Data. Social Networks. 1988;10:359–382.

[62] Borenstein M, Hedges LV, Higgins JPT, Rothstein HR. Introduction to Meta-Analysis. Chichester, United Kingdom: John Wiley and Sons Ltd; 2009.

[63] DerSimonian R, Laird N. Meta-analysis in clinical trials. Controlled Clinical Trials. 1986;7(3):177–188. 3802833 10.1016/0197-2456(86)90046-2

[64] Fisher RA. Frequency distribution of the values of the correlation coefficient in samples of an indefinitely large population. Biometrika. 1915;10(4):507–521.

[65] Fisher RA. On the’probable error’ of a coefficient of correlation deduced from a small sample. Metron. 1921;1:3–32.

[66] Inglehart R, Welzel C. Changing Mass Priorities: The Link between Modernization and Democracy. Perspectives on Politics. 2010;8:551–567.

[67] Verkuyten M, Hagendoorn L, Masson K. The Ethnic Hierarchy among Majority and Minority Youth In The Netherlands. Journal of Applied Psychology. 1996;26:1104–1118.

[68] Smith S. Ethnic Segregation in Friendship Networks. Studies of its Determinants in English, German, Dutch, and Swedish School Classes. Utrecht University Utrecht, Netherlands; 2015.

[69] Nyen T, Gravdahl HP, Eidset I. Valg av utdanning er en prosess. Bergen, Norway: Norwegian Ministry of Social Issues and Health; 2000.

[70] Panican A. Väljer unga fel?—grundskoleelevers attityder till gymnasievalet. Ratio and Lund University; 2015. Available from: http://ratio.se/app/uploads/2015/06/valjer-unga-fel_ungas-attityder-till-gymnaasievalet.pdf.

[71] Lund S. Marknad och medborgare: elevers valhandlingar i gymnasieutbildningens integrations- och differentieringsprocesser. Växjö University. Växjö; 2006.

[72] Bursell M, Jansson F. Diversity preferences among employees and ethnoracial workplace segregation. Social Science Research. 2018;74:62–76. 29961490 10.1016/j.ssresearch.2018.03.009

[73] Jansson F, Bursell M. Social consensus influences ethnic diversity preferences. Social Influence. 2018;13(4):192–208.

[74] Leckie G, Goldstein H. The Limitations of Using School League Tables to The Limitations of Using School League Tables to Inform School Choice. Journal of the Royal Statistical Society. 2009;172(4):835–851.

