# Supplementary material for: Segregation within school classes: Detecting social clustering in choice data
Source: PLoS One. 2020 Jun 1;15(6):e0233677. doi: 10.1371/journal.pone.0233677 (PMC7263592; doi:10.1371/journal.pone.0233677)
Supplement: Peer review history

# Peer review: Decision Letter 0

**DOI:** 10.1371/journal.pone.0233677.r001

**Authors:** Ronald Breiger

10 Feb 2020

PONE-D-19-35338

Segregation within school classes:

Deriving social clustering from choice data

PLOS ONE

Dear Dr. Jansson,

Thank you for submitting your manuscript to PLOS ONE. After careful consideration, we feel that it has merit but does not fully meet PLOS ONE’s publication criteria as it currently stands. Therefore, we invite you to submit a revised version of the manuscript that addresses the points raised during the review process.

We would appreciate receiving your revised manuscript by Mar 26 2020 11:59PM. When you are ready to submit your revision, log on to https://www.editorialmanager.com/pone/ and select the 'Submissions Needing Revision' folder to locate your manuscript file.

If you would like to make changes to your financial disclosure, please include your updated statement in your cover letter.

To enhance the reproducibility of your results, we recommend that if applicable you deposit your laboratory protocols in protocols.io, where a protocol can be assigned its own identifier (DOI) such that it can be cited independently in the future. For instructions see: http://journals.plos.org/plosone/s/submission-guidelines#loc-laboratory-protocols

Please include the following items when submitting your revised manuscript:

- A rebuttal letter that responds to each point raised by the academic editor and reviewer(s). This letter should be uploaded as separate file and labeled 'Response to Reviewers'.
- A marked-up copy of your manuscript that highlights changes made to the original version. This file should be uploaded as separate file and labeled 'Revised Manuscript with Track Changes'.
- An unmarked version of your revised paper without tracked changes. This file should be uploaded as separate file and labeled 'Manuscript'.

Please note while forming your response, if your article is accepted, you may have the opportunity to make the peer review history publicly available. The record will include editor decision letters (with reviews) and your responses to reviewer comments. If eligible, we will contact you to opt in or out.

We look forward to receiving your revised manuscript.

Kind regards,

Ronald Breiger, Ph.D.

Academic Editor

PLOS ONE

Additional Editor Comments (if provided):

Let us suppose that there are n students and c countries of origin. Construct the binary affiliation matrix ("two-mode matrix"), call it F, that is of size (n rows, c columns) and that has a "1" if the student in that row is from the country in that column. Using ordinary (inner-product) matrix multiplication (*), we may multiply F by its transpose (F^T, the latter being the matrix with c rows and n columns in which the rows in F are the columns of F^T): F * F^T, which is a matrix with n rows and n columns, reporting whether the student in row i shares a country of origin with the student in row j. This matrix (F * F^T), binarized if necessary, is, I believe, your matrix A, which you introduce on page 9, lines 227-236. Similar comments apply to your matrix B (introduced in the same paragraph), which compares the students to one another based on whether each student shares the choice of the same curricular track (given by a second affiliation matrix, one with n rows and t columns if there are t tracks in all). Then, much of your paper concerns relations among the two square matrices, A and B, using the network-analytic procedure, QAP. I think that indicating that the underlying data for much of your paper are two sets of affiliation (two-mode binary) matrices would help avoid confusion such as that of Reviewer 2 who did not understand why you write (p. 10, lines 279-280) that "it is not even possible to have exactly two ties in a triad.” The reason that you are correct is that if, for example, students 2 and 3 choose the same curricular track as does student 1, then all pairs of students 1, 2, and 3 would be connected (by virtue of their common membership in the same curricular track).

I am much more sympathetic with Reviewer 2 on many other points. Your frequent use of the word "choices" is confusing because, in much of the literature on social networks, "choices" are given in a person-to-person ("one-mode") matrix in which, in principle, anyone might choose anyone else (for example: a matrix of friendship "choices," very different than the case for your matrices A and B defined above which derive from affiliation matrices -- *if* I am understanding your procedure correctly). You do state your definition of "choices" well, and clearly ("We will explore similarities in ... choices of specialisation tracks...."; p. 2, lines 33-34). I would ask you to try to find a way to make clear to the reader that your use of the word "choices" refers to "choice of curricular track," and (specifically) does not refer to "choices" such as "friendship choices in one-mode networks," as I think many readers would naturally (if incorrectly) assume. Indeed, I think that, often, when you use the word "choices" you can rewrite it as "choices of curricular track," and that this would help to avoid much confusion. More generally, Reviewer 2 asks you to clarify your thinking about homogeneity vs. peer effects, concentration vs. composition, endogenous vs. exogenous, contagion vs. social network ties based on preference or avoidance for connections with specific others in one's social network. I agree that clarifying (where necessary) your position on these issues would help you to address the concern of Reviewer 2 regarding the clarity of your overall contribution.

I agree with Reviewer 1 that there is much to like about your paper, and with Reviewer 2 that your paper is interesting and potentially innovative. Within the overall positive framework of Reviewer 1's comments, the reviewer nonetheless asks you to consider qualifying the degree of claimed generality of the analytical framework presented in this paper. Indeed, some of Reviewer 1's comments (see the reference to a classroom that might be 50% girls and 50% boys, and why a single girl would be expected to be peer-influenced by every other girl) are similar to those of Reviewer 2 in that they encourage you to clarify the relation between your networks based on affiliation ties, and social network ties (or what Reviewer 1 calls "meaningful social relations" as opposed to shared affiliations in a curricular track). Concerns about the relation of homophily to peer influence are raised here as well. And, in what ways might your method be generalized to apply to settings other than schools?

I agree with both reviewers that your project is worthy and innovative, and in encouraging you to revise your paper in a way that takes account of their comments to improve the clarity of your presentation of your contributions. For points that you may disagree with, please explain your point of view on those, in a separate memo. (Minor: on page 28, line 750, I think you mean MMC, not MCC.)

Journal Requirements:

When submitting your revision, we need you to address these additional requirements:

1. Please ensure that your manuscript meets PLOS ONE's style requirements, including those for file naming. The PLOS ONE style templates can be found at http://www.plosone.org/attachments/PLOSOne_formatting_sample_main_body.pdf and http://www.plosone.org/attachments/PLOSOne_formatting_sample_title_authors_affiliations.pdf

2. We note that you have indicated that data from this study are available upon request. PLOS only allows data to be available upon request if there are legal or ethical restrictions on sharing data publicly. For more information on unacceptable data access restrictions, please see http://journals.plos.org/plosone/s/data-availability#loc-unacceptable-data-access-restrictions.

In your revised cover letter, please address the following prompts:

a) If there are ethical or legal restrictions on sharing a de-identified data set, please explain them in detail (e.g., data contain potentially sensitive information, data are owned by a third-party organization, etc.) and who has imposed them (e.g., an ethics committee). Please also provide contact information for a data access committee, ethics committee, or other institutional body to which data requests may be sent.

b) If there are no restrictions, please upload the minimal anonymized data set necessary to replicate your study findings as either Supporting Information files or to a stable, public repository and provide us with the relevant URLs, DOIs, or accession numbers. For a list of acceptable repositories, please see http://journals.plos.org/plosone/s/data-availability#loc-recommended-repositories.

We will update your Data Availability statement on your behalf to reflect the information you provide.

3. Your ethics statement must appear in the Methods section of your manuscript. If your ethics statement is written in any section besides the Methods, please move it to the Methods section and delete it from any other section. Please also ensure that your ethics statement is included in your manuscript, as the ethics section of your online submission will not be published alongside your manuscript.

[Note: HTML markup is below. Please do not edit.]

Reviewers' comments:

Reviewer's Responses to Questions

**Comments to the Author**

1. Is the manuscript technically sound, and do the data support the conclusions?

The manuscript must describe a technically sound piece of scientific research with data that supports the conclusions. Experiments must have been conducted rigorously, with appropriate controls, replication, and sample sizes. The conclusions must be drawn appropriately based on the data presented. 

Reviewer #1: Yes

Reviewer #2: Partly

**********

2. Has the statistical analysis been performed appropriately and rigorously? 

Reviewer #1: Yes

Reviewer #2: Yes

**********

3. Have the authors made all data underlying the findings in their manuscript fully available?

The PLOS Data policy requires authors to make all data underlying the findings described in their manuscript fully available without restriction, with rare exception (please refer to the Data Availability Statement in the manuscript PDF file). The data should be provided as part of the manuscript or its supporting information, or deposited to a public repository. For example, in addition to summary statistics, the data points behind means, medians and variance measures should be available. If there are restrictions on publicly sharing data—e.g. participant privacy or use of data from a third party—those must be specified.

Reviewer #1: Yes

Reviewer #2: No

**********

4. Is the manuscript presented in an intelligible fashion and written in standard English?

PLOS ONE does not copyedit accepted manuscripts, so the language in submitted articles must be clear, correct, and unambiguous. Any typographical or grammatical errors should be corrected at revision, so please note any specific errors here.

Reviewer #1: Yes

Reviewer #2: No

**********

5. Review Comments to the Author

Please use the space provided to explain your answers to the questions above. You may also include additional comments for the author, including concerns about dual publication, research ethics, or publication ethics. (Please upload your review as an attachment if it exceeds 20,000 characters)

Reviewer #1: This paper offers a method for inferring peer effects on choices using large-scale register data of behavior/revealed preferences. The key challenge is to separate these endogenous preferences (i.e., being influenced by others) from common exogenous preferences (related to, e.g., ethnicity, gender, or social class). This challenge is intend to be met by comparing choices within- and between cohorts in the same school. After explaining the method, it is illustrated with Norwegian register data. The key is that having the same country of origin correlates with making similar educational choices within schools.

There is much to like about this paper. To begin with, it addresses an important methodological problem, and its suggested method to address this problem is innovative and potentially useful. The method itself is explained sufficiently and understandably. The empirical analysis is convincing, thus providing a nice example of how the method can be applied. All steps of the analysis are well-explained, and many looming questions are answered on spot. The results section also is fine, and it is great that effect size is discussed, that categorical dimensions are also considered, and several robustness checks are reported.

With this overall positive impression in mind, I have several comments, which are ultimately related and come down to the question whether the proposed method is indeed as “general” as the abstract claims. In addition, my impression is that in its current version, the paper is a mix between a (more general) methodological and a (more specific) substantive contribution.

To be sure, it is of course fine to claim both contributions (though, as explained below, I am not fully convinced about the more general applicability of the suggested method). But I fear that swinging back and forth between its substantive and its methodological contribution might ultimately weaken the paper on both fronts. Specifically, large parts of the introduction, almost the complete theory section, and most of the discussion/conclusion discuss research on segregation and peer effects in school. On the other hand, a novel method is proposed, and it is explicitly said (e.g., on p. 6) that it is the “aim to make a methodological contribution.” Surprisingly, though, neither the discussion nor the conclusion has much to say about this methodological contribution. Instead, there is a quite lengthy substantial discussion of the findings.

Importantly, however, this is *not* (just, or even mainly) a question of how to frame the paper. In fact, I am not yet convinced that the method is generally suitable for “deriving social clustering from choice data”. For starters, the suggested method crucially rests on the assumption that students of the same ethnic origin actually engage in meaningful interaction on the ground. As is argued in the paper, because of ethnic homophily, this assumption seems reasonable. But doesn’t this at the same time considerably limit the usefulness of the method for studying dimensions other than ethnicity? For instance, gender is mentioned as another dimension of similarity. Yet while gender structures ties among students, unlike ethnicity, in most schools gender is more or less equally distributed. But in classrooms with 50% boys and 50% girls it is far from clear why a single girl would be “peer influenced” by all other girls, which is what the method would suggest/test. (As a side note, this is why I was not surprised that “most of the effect for gender seems to be explained by common exogenous preferences” (p. 22). I suspect that this is because the proposed method does not cover meaningful social interaction for girls.)

In other words, the method seems to depend on the assumption that similarity (e.g., ethnic origin or gender) actually translates into meaningful social relations in class. Again, this might apply to ethnicity, but for many other dimensions this assumption is questionable. So an important question is whether the suggested method indeed is “general”, as said in the abstract, or whether its applicability is not limited to dimensions for which a reasonable large amount of homogeneity in ties can be assumed?

A related issue is relative group size. On p. 16 the main hypothesis is that students with the same country of origin will influence each other’s choices. Fair enough, but doesn’t it make a huge difference if there are, say, two Iraqi children in a school class or ten? Is it really plausible to assume that a single Iraqi kid will be influenced by the one other Iraqi kid in class, and not by any of her other inter-ethnic peers, many of which of them probably are her friends? I am not saying that this needs to be addressed in additional analysis (though of course it would be great if this could be accounted for). But some reflection on this point would be very welcome.

Still related is the context of schools, which also, I think, more fundamentally limits the methods than is currently admitted in the paper. The key idea of the proposed method is to test the impact of social exposure and exogenous effects by comparing students not to their classmates but to other students in the same programme and school, who started in a different year (p. 9). This makes sense, but it also involves quite strong assumptions that are currently not mentioned/stressed enough. For example, this only works if there is no time-heterogeneity, i.e., if cohorts from different years do not differ in terms of the exogenous effects. This is even hinted at, as it is said on p. 14 that the number of cohorts is limited to five years “to maintain similarity for the between-cohort comparisons”. Still, this assumption is so crucial that it should be clearly stated and discussed. (Also, alternative specifications might be tested and reported in the appendix, e.g., using only three years instead of five.)

Next, what if students are influenced by older peers in school? On p. 2 it is in fact said that exogenous preferences are likely to be influenced by, among others, previous teachers and older students. But older students are obviously in different cohorts. Perhaps this issue could be addressed by comparing students not to any other cohort but only to younger cohorts? For example, not, as given as an example on p. 10 comparing students starting in 2006 with to students starting in 2007-2011, but comparing students in 2011 with students starting in 2006-2010.

Finally, the overall reasoning of the method is legit for schools, but what about other social contexts? Many other contexts such as neighborhoods, organizations, firms etc. are much less easily separable into cohorts. On p. 26 it is indeed said that the method could be applied “when it is possible to do both within- and between-cohort comparisons.” But when is this the case? Some examples would help. Especially because on the same page, “mobility within jobs or between firms, marriage decisions and families’ decisions on where to move” are mentioned, but it is far from clear how the current method could be applied in these settings.

(Note that I assume that the general methodological contribution is the main aim of the paper; if I am mistaken and the substantive contribution is the more important one, some of my concerns outlined above might be neglectable.)

Minor points and suggestions:

- For readers who are not familiar with network analysis/adjacency matrices, a figure/illustration of the two matrices might be helpful.

- Consider replacing Table 1 by a figure, as this would allow comparing results more easily.

Reviewer #2: After close and repeated readings, I believe the main point of this paper can be summarized as follows: There are two reasons similar students in the same class might make similar choices: One is because of their similar backgrounds, and one is because they have influenced each other. And one way we can separate these two reasons is by comparing the choices of similar students within and across classrooms—in other words, students who share both a common social setting AND background traits, on one hand, with students who only share background traits, on the other.

IF my above summary is accurate, this paper seems to provide an interesting (and potentially even innovative) way of identifying an effect that is challenging to interpret. The second, shorter part of my critique will focus on the latter point (that the effect is challenging to interpret). The first part of my critique will focus on the big “IF” above: It was challenging for me to figure out what this paper is essentially about (which of course made it difficult to assess it).

CLARITY

It was very hard for me to determine the authors’ basic aims. Based on the title—and supported by the abstract—I expected the authors to introduce a new approach for identifying the attributed-based “clustering” (i.e. segregation) of network ties. Later, it seemed that what the authors were trying to do was identify evidence of both clustering AND peer influence in their data (this impression was encouraged especially in the section entitled “Causes and effects of segregation in school”—two very different classes of social phenomena that are discussed interchangeably). And only later did I come to understand what is actually (I think) going on here: The authors identify evidence of peer influence, ASSUMING that homophily is part of the cause.

It wasn’t until I got to the results section that this finally became clear to me. I would strongly recommend that the authors revisit the bigger picture and try to be very clear from the outset what they are trying to demonstrate (and how) and articulate the flow of their argument more clearly (in particular, what is actually demonstrated and what is assumed). There are two additional issues that don’t help things. First is a problem of language. For instance, terms like “social effects” and “social choice” are used by the authors in a very precise way that is not entirely intuitive. (These are words that, out of context, are a priori already fairly confusing. For instance: “In particular, we have explored if students make more similar choices to peers of the same country of origin as compared to other students and whether this is a social choice.”) The authors use “exogenous” and “endogenous” in a very specific way that wasn’t immediately clear. “Clustering” seems to be used to refer both to CURRENT segregation and to the intention of students to cluster together in the FUTURE; “ties” (and even “graph structure”) don’t really refer to ties/graphs at all in the sense of actual social connections (but rather, similarity matrices among students); and the term “social educational choices” is a poor way of summarizing “choices about my education that have been influenced by my peers.”

Second, there is a problem of very specific excerpts that don’t really make sense and claims that I don’t believe are well-founded. Here are just a handful of examples:

On p. 2, “ethnic school segregation is usually measured as the concentration of immigrant peers at school level, or within educational cohorts within schools.” How is this different from the ethnic COMPOSITION of a school/cohort? Also on that page, “yet there were few quantitative studies on whether and how students interact at school.” This seems to suggest there are few studies of student social networks, which is not at all the case (in fact, the authors cite many of them).

On p. 3, “our main research question is whether we may infer homophily effects on students’ educational choices,” where “homophily” appears to mean “peer influence specifically from homophilous ties.” This is not at all how “homophily” is usually used. (Specifically, homophily explains variation in the patterning of network ties, not variation in individuals’ outcomes.)

On p. 6, the authors refer to prior literature on social networks using name generators as if these were “stated preferences” and liken their own approach to documenting “revealed preferences” (for intra-ethnic interaction). But identifying who my friends are does not strike me at all as an example of stated ethnic preferences (because, notwithstanding measurement error, these refer to actual social ties, and so more closely seem like revealed preferences) and the authors’ approach doesn’t “reveal” intra-ethnic preferences at all (rather, at best they demonstrate the consequences of segregation coupled with peer influence—where segregation could be caused by any number of factors, not just intra-ethnic preferences).

Finally, on p. 10: “Given students A, B and C, if both B and C have made the same choice as A, then we know that B and C must also have made the same choice. This is the extreme, deterministic, case of a triad closure: it is not even possible to have exactly two ties in a triad.” The first sentence seems tautological; the second doesn’t make sense at all. (Why can’t there be an “open” triad with just two sides, and what does this have to do with the first sentence?)

Again, I think that much of my confusion stems from the inherently somewhat complex and ambiguous objective of this paper: To document the effects of peer influence, ASSUMING students with similar backgrounds are affiliated with one another. The authors need to find a way to make both their objectives and their assumptions—and thus the exact nature of their argument—clearer. Eliminating confusing language and specific excerpts (like those above) will also help.

INTERPRETATION

That said, even with clearer language and a tighter text, the goal of this paper is challenging to explain, precisely BECAUSE it is so complex and the results so tough to interpret. Insofar as students from the same class DO share similar choices—more so than they share similar choices with similar students from different classes—this plausibly derives from peer influence. But, as the authors note, it would be much better to deal with a choice outcome that didn’t ALSO involve the students spending more time together; whereas here, it is impossible to distinguish the two (i.e. the social “contagion” of your choice impacting mine versus me making the same choice as you SO THAT we can spend more time together). What if students are not mimicking each other—but making the opposite selections of other classmates they don’t like and want to avoid? The assumption of homophily is also somewhat problematic. Surely there are a large number of inter-group ties that are also conducive to peer influence; and the density of in-group ties might itself vary (so, in a sense, weak homophily combined with strong peer influence could generate the same consequence as strong homophily combined with weak peer influence). It may also be the case that I make similar choices to other students like me, not because we are friends, but because they serve as a reference group (i.e. I want to imitate people “like me”).

At the end of the day, then, it seems that what the authors are able to identify is something of a black box of unexplained variation—one that probably entails both homophily and peer influence at work, but it’s impossible to be very precise about either (and could also result from other factors). Consequently, the authors’ conclusions are also appropriately qualified: “This is not likely to be exclusively an effect of having a common background as such… we believe the simplest explanation to be an increased social exchange between the students.” This is not entirely persuasive—especially when there are many other methods available for documenting both homophily and influence, as well as separating the two. Underscoring this approach’s unique contribution would be helpful.

**********

6. PLOS authors have the option to publish the peer review history of their article (what does this mean?). If published, this will include your full peer review and any attached files.

If you choose “no”, your identity will remain anonymous but your review may still be made public.

**Do you want your identity to be public for this peer review?** For information about this choice, including consent withdrawal, please see our Privacy Policy.

Reviewer #1: No

Reviewer #2: No

[NOTE: If reviewer comments were submitted as an attachment file, they will be attached to this email and accessible via the submission site. Please log into your account, locate the manuscript record, and check for the action link "View Attachments". If this link does not appear, there are no attachment files to be viewed.]

While revising your submission, please upload your figure files to the Preflight Analysis and Conversion Engine (PACE) digital diagnostic tool, https://pacev2.apexcovantage.com/. PACE helps ensure that figures meet PLOS requirements. To use PACE, you must first register as a user. Registration is free. Then, login and navigate to the UPLOAD tab, where you will find detailed instructions on how to use the tool. If you encounter any issues or have any questions when using PACE, please email us at figures@plos.org. Please note that Supporting Information files do not need this step.

# Author comment: Author response to Decision Letter 0

**DOI:** 10.1371/journal.pone.0233677.r002

16 Apr 2020

We would like to thank both the editor and the reviewers for insightful and constructive comments. Almost all comments have led to revisions of the manuscript, and we hope that it has now become clearer what the purpose is, what conclusions can be drawn and how to interpret the results, and what are the general requirements for the method. We answer the specific comments directly in the attached document, Response to Reviewers.

Attachment Submitted filename: Response to Reviewers.pdf Click here for additional data file.

# Editor report: Decision Letter 1

**DOI:** 10.1371/journal.pone.0233677.r003

**Authors:** Ronald Breiger

12 May 2020

Segregation within school classes: Detecting social clustering in choice data

PONE-D-19-35338R1

Dear Dr. Jansson,

We are pleased to inform you that your manuscript has been judged scientifically suitable for publication and will be formally accepted for publication once it complies with all outstanding technical requirements.

Within one week, you will receive an e-mail containing information on the amendments required prior to publication. When all required modifications have been addressed, you will receive a formal acceptance letter and your manuscript will proceed to our production department and be scheduled for publication.

Shortly after the formal acceptance letter is sent, an invoice for payment will follow. To ensure an efficient production and billing process, please log into Editorial Manager at https://www.editorialmanager.com/pone/, click the "Update My Information" link at the top of the page, and update your user information. If you have any billing related questions, please contact our Author Billing department directly at authorbilling@plos.org.

If your institution or institutions have a press office, please notify them about your upcoming paper to enable them to help maximize its impact. If they will be preparing press materials for this manuscript, you must inform our press team as soon as possible and no later than 48 hours after receiving the formal acceptance. Your manuscript will remain under strict press embargo until 2 pm Eastern Time on the date of publication. For more information, please contact onepress@plos.org.

With kind regards,

Ronald Breiger, Ph.D.

Academic Editor

PLOS ONE

Additional Editor Comments (optional):

The reviewers of your original submission provided a number of appropriate and helpful comments and criticism of that submission. I have read your revised submission with great care, and I find that you have revised your manuscript in ways that have transformed a very important original submission into a substantially improved revised version. I found the clarity of the revised version to be greatly enhanced, not only with respect to language, and the precision of the definitions of your major concepts, but also with reference to describing with great clarity the soundness of your technical approach. You have made excellent use of the reviewers' comments. I think your (revised) manuscript presents an exemplary work of importance concerning both the substantive analysis and the general method, an excellent contribution. I especially appreciated the care that went into your "Data Requirements" section, the addition of sections on ethics, presentation of the issue of data availability, and the provision (via github) of a well-documented suite of programs and an example in R.

Reviewers' comments:
